# RNF8 up-regulates AR/ARV7 action to contribute to advanced prostate cancer progression

**DOI:** 10.1038/s41419-022-04787-9

**Published:** 2022-04-15

**Authors:** Tingting Zhou, Shengli Wang, Xiaoyu Song, Wensu Liu, Fang Dong, Yunlong Huo, Renlong Zou, Chunyu Wang, Siyi Zhang, Wei Liu, Ge Sun, Lin Lin, Kai Zeng, Xiang Dong, Qiqiang Guo, Fei Yi, Zhuo Wang, Xiaoman Li, Bo Jiang, Liu Cao, Yue Zhao

**Affiliations:** 1 Department of Cell Biology, Key Laboratory of Cell Biology, Ministry of Public Health, and Key Laboratory of Medical Cell Biology, Ministry of Education, School of Life Sciences, China Medical University, Shenyang, Liaoning Province 110122 P. R. China; 2 College of Basic Medical Science, Institute of Health Sciences, Key Laboratory of Cell Biology of Ministry of Public Health, Key Laboratory of Medical Cell Biology of Ministry of Education, Liaoning Province Collaborative Innovation Center of Aging Related Disease Diagnosis and Treatment and Prevention, China Medical University, Shenyang, Liaoning Province 110122 P. R. China; 3 Department of Pathology, Shengjing Hospital of China Medical University, Shenyang, Liaoning Province 110004 P. R. China

**Keywords:** Prostate cancer, Transcription

## Abstract

Androgen receptor (AR) signaling drives prostate cancer (PC) progression. Androgen deprivation therapy (ADT) is temporally effective, whereas drug resistance inevitably develops. Abnormal expression of AR/ARV7 (the most common AR splicing variant) is critical for endocrine resistance, while the detailed mechanism is still elusive. In this study, bioinformatics and immunohistochemical analyses demonstrate that RNF8 is high expressed in PC and castration-resistant PC (CRPC) samples and the expression of RNF8 is positively correlated with the Gleason score. The high expression of *RNF8* in PCs predicts a poor prognosis. These results provide a potential function of RNF8 in PC progression. Furthermore, the mRNA expression of *RNF8* is positively correlated with that of *AR* in PC. Mechanistically, we find that RNF8 upregulates c-Myc-induced *AR* transcription via altering histone modifications at the c-Myc binding site within the *AR* gene. RNF8 also acts as a co-activator of AR, promoting the recruitment of AR/ARV7 to the *KLK3* (*PSA*) promoter, where RNF8 modulates histone modifications. These functions of RNF8 are dependent on its E3 ligase activity. RNF8 knockdown further reduces AR transactivation and PSA expression in CRPC cells with enzalutamide treatment. RNF8 depletion restrains cell proliferation and alleviates enzalutamide resistance in CRPC cells. Our findings indicate that RNF8 may be a potential therapeutic target for endocrine resistance in PC.

## Introduction

Prostate cancer (PC) is the second most prevalent malignancy in men worldwide [1]. As a ligand-dependent transcription factor (TF), androgen receptor (AR) is essential for normal prostate epithelial growth and maintenance. AR signaling is persistently active during PC progression. Thus, androgen deprivation therapy (ADT) is the most effective treatment for PC. However, the effect is temporary. The incurable castration-resistant prostate cancer (CRPC) frequently emerges and becomes a treatment obstruction [2].

Enzalutamide, a potent AR antagonist, has been approved to treat CRPC by the US Food and Drug Administration (FDA) [3, 4]. Although enzalutamide significantly prolongs the survival of CRPC patients, drug resistance inevitably occurs [5, 6]. The relapse of PC after ADT and the resistance to enzalutamide are mainly associated with AR amplification, ligand-independent AR splice variants (AR-Vs), aberrant expression of co-regulators, and proliferation pathways activation [5–10]. Nevertheless, only 10–20% of CRPC patients with overexpressed AR carry amplification of the *AR* gene [7], suggesting that other mechanisms upregulate AR expression. ARV7, as one of the most common AR-Vs, exhibits increased expression in CRPC patients [11]. High expression of AR and ARV7 is closely associated with androgen-independent PC progression [12–14]. Moreover, several AR co-regulators with abnormal expression in PC can reactivate AR signaling to promote PC progression [15–18]. However, the underlying mechanisms contributing to AR/AR-Vs overexpression and AR signaling reactivation remain enigmatic and need further study.

RING finger E3 ligases are frequently reported to be involved in cancer development with various mechanisms [19–22], appealing to concern about this family regarding carcinogenesis. RNF8, as a RING finger E3 ligase, regulates histone H2A and H2B ubiquitination (uH2A and uH2B) involved in transducing DNA double-strand breaks (DSBs) signals [23, 24]. RNF8 also functions in transcriptional regulation, cell cycle regulation, and tumorigenesis [25–35]. Recent studies have also reported the positive role of RNF8 in promoting tumor progression [30, 31, 34, 36–39]. We have previously identified that RNF8 regulates the activity of ERα through protein stability and acts as an ERα co-factor promoting breast cancer proliferation [35]. However, whether RNF8 participates in the progression of PC by regulating AR activity is currently unknown.

To broaden the comprehension of the mechanism of PC progression, we identify that RNF8 acts as a ligand-independent activator of AR/ARV7. High expression of RNF8 predicts poor survival in PC patients. The expression of RNF8 is high in clinical PC samples and positively correlated with the Gleason score (GS) of PC. Moreover, RNF8 is high expressed in AR-positive PC and CRPC cell lines. The mRNA expression of *RNF8* is positively correlated with that of *AR* in PC. These findings suggest the role of RNF8 in regulating AR signaling and PC progression. Mechanically, RNF8 enhances *AR* transcription via forming a complex with c-Myc and modulates the histone modifications at the c-Myc binding site in the *AR* gene. Unexpectedly, RNF8 also acts as a co-activator of AR, promoting AR/ARV7 recruitment to the AR response element (ARE) of the *KLK3* (*PSA*) promoter and modulating histone modifications at the ARE to regulate the transcription of various AR target genes. Silencing RNF8 further reduces AR transactivation and PSA expression in CRPC cells treated with enzalutamide. Furthermore, RNF8 knockdown retards the proliferation of AR-positive PC cells and sensitizes CRPC cells to enzalutamide.

Collectively, our results identify RNF8 as an activator of the AR/ARV7 ligand independently. Our findings imply an essential role of RNF8 in the progression of PC and ADT therapy resistance.

## Results

### The expression of RNF8 is increased in advanced prostate cancer and positively correlated with that of AR

**Fig. 1:**
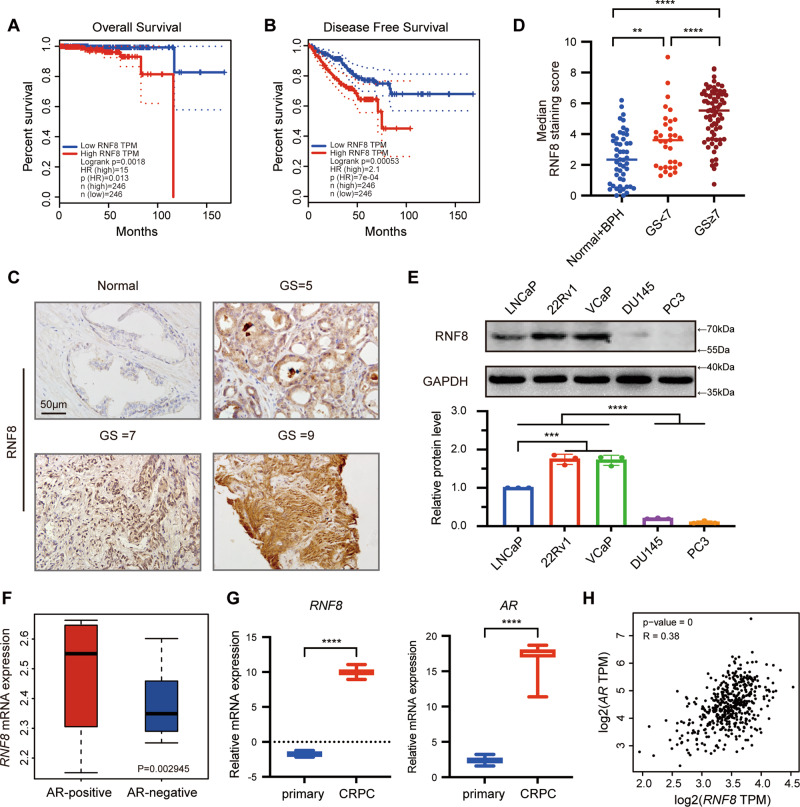
The expression of RNF8 is increased in advanced prostate cancer and positively correlated with that of AR. **A**, **B** Kaplan–Meier analysis of overall survival (**A**) and disease-free survival (**B**) of PC patients (*n* = 246) with low or high expression of RNF8 in TCGA. **C** Representative images of RNF8 immunohistochemical staining in normal prostate and PC tissues. Scale bar, 50 μm. **D** Statistical analysis of the median staining score of RNF8 in normal prostate (*n* = 14), BPH (*n* = 36), and PC tissues (*n* = 105). **P < 0.01; ****P < 0.0001 (two-sided Mann–Whitney test). **E** Upper: the protein levels of RNF8 in LNCaP, 22Rv1, VCaP, DU145, and PC3 cells were determined by western blot. Lower: the result is presented as the relative gray value normalized to GAPDH (means ± SEM, *n* = 3). ***P < 0.001; ****P < 0.0001 (One-way ANOVA). **F** Quantification analysis of *RNF8* mRNA expression in AR-positive (*n* = 286) and AR-negative (*n* = 273) PC. *P* = 0.002945 (two-sided unpaired *t*-test). **G** Quantification analysis of *RNF8* and *AR* mRNA expression in primary PC (*n* = 11) and CRPC tissues (*n* = 45). ****P < 0.0001 (two-sided unpaired *t*-test). **H** Scatter plot analysis of the correlation be*t*ween *RNF8* versus *AR* mRNA expression in PC was determined by Pearson correlation coefficient analysis. *P* = 0, *R* = 0.38.

Based on microarray staining data from The Human Protein Atlas, 75% of the PC samples show high/medium expression of RNF8, which is the highest staining frequency among several cases of cancers [40]. The survival analysis by GEPIA [41] exhibits that *RNF8* mRNA expression is inversely correlated with the overall and disease-free survival outcomes (Fig. 1A, B). Then, we evaluated the impact of clinicopathological characteristics and *RNF8* mRNA expression on the overall survival (OS) of PC patients by COX regression analysis (Supplementary Table 1). The results indicated that RNF8 is not an independent predictor of OS. Whereas, a Jonckheere–Terpstra test for ordered alternatives demonstrated that there was a statistically significant trend of higher median *RNF8* mRNA expression with higher GS (from “<7”, “=7”, to “>7”) (Supplementary Table 2). The percentage of positive staining of RNF8 increased in PC tissues compared with that in normal prostate and BPH tissues (Fig. 1C, D, Supplementary Fig. 1A, B, and Table 3), and RNF8 expression exhibited a positive trend with GS (Supplementary Table 2).

Compared to AR-negative PC cells (DU145 and PC3), RNF8 was dominantly expressed in AR-positive ones (LNCaP, 22Rv1, and VCaP) (Fig. 1E). RNF8 expression was higher in CRPC cells (22Rv1 and VCaP) than that in androgen-dependent cells (LNCaP) (Fig. 1E). Analysis from GEO data (GSE86532, GSE110903) [42] demonstrated that *RNF8* mRNA expression was higher in AR-positive than that in AR-negative PC samples (Fig. 1F). GEO data (GSE74367) [43] analysis exhibited that both *RNF8* and *AR* mRNA expressions were increased in CRPC compared with that in primary PC (Fig. 1G). Furthermore, it was shown that *RNF8* mRNA expression is positively correlated with *AR* mRNA expression in PC samples analyzed by GEPIA (Fig. 1H).

The above results indicate a positive relationship between RNF8 and AR in PC progression.

### RNF8 enhances *AR* transcription dependent on its E3 ligase activity

**Fig. 2:**
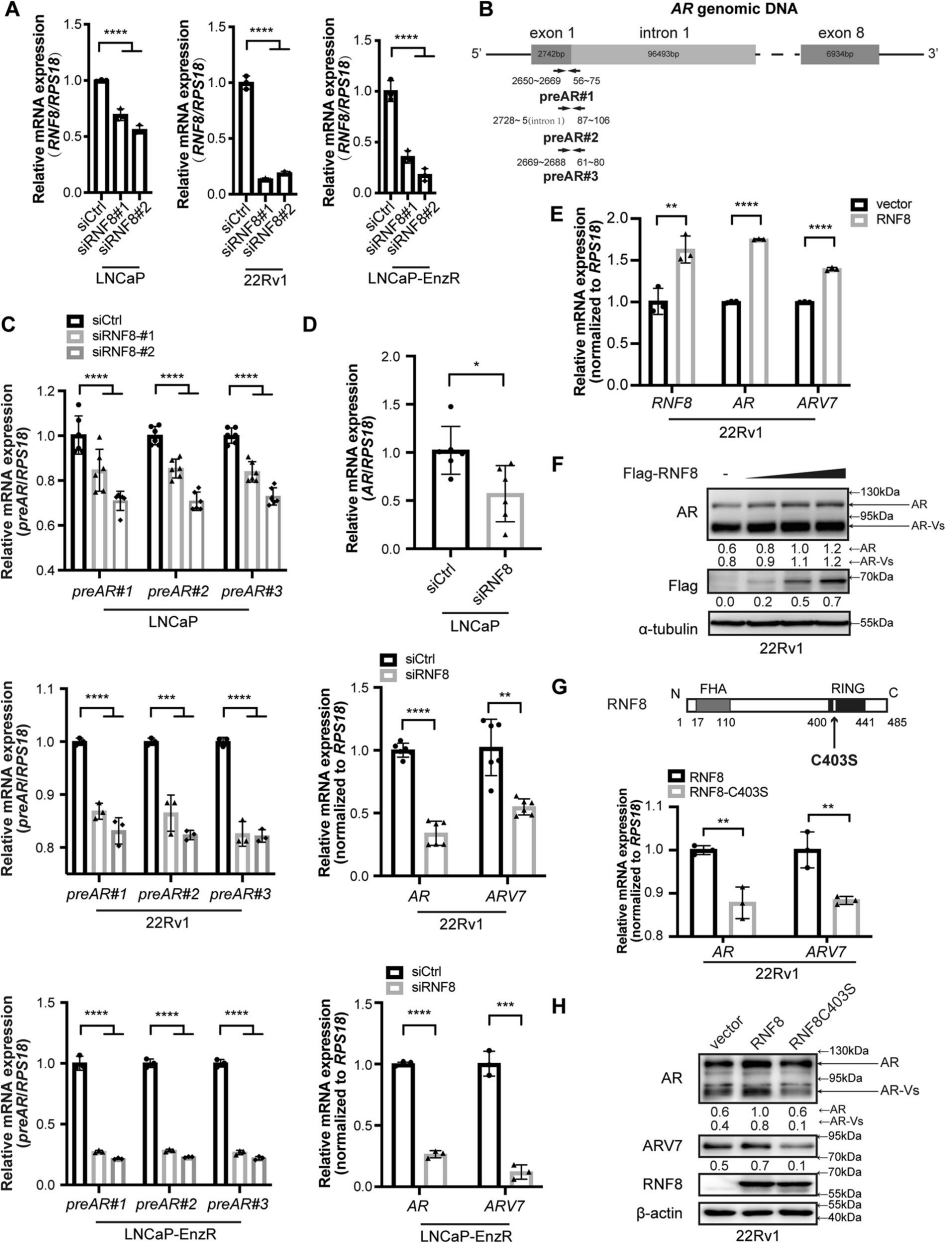
RNF8 enhances *AR* transcription dependent on its E3 ligase activity. **A** The efficacy of siRNA targeting RNF8 in LNCaP, 22Rv1, and LNCaP-EnzR cells was determined by qRT-PCR (*n* = 3). **B** Histogram of primer binding sites on *AR* pre-mRNA based on *AR* genomic DNA sequence. **C** The transcription levels of *AR* pre-mRNA were detected by three pairs of primers in control and RNF8 silencing LNCaP (*n* = 6), 22Rv1 (*n* = 3), and LNCaP-EnzR (*n* = 3) cells. **D** The transcription levels of *AR* and *ARV7* in control and RNF8 silenced LNCaP (*n* = 6), 22Rv1 (*n* = 6), and LNCaP-EnzR (*n* = 3) cells. **E** The transcription levels of *AR* and *ARV7* in 22Rv1 cells with RNF8 overexpression (*n* = 3). **F** The protein levels of AR and AR-Vs in RNF8 overexpressed 22Rv1 cells were determined by western blot. **G** Upper: schematic representation of RNF8 ligase inactivation point mutation (RNF8C403S). Numbers indicate amino acid positions. Lower: the mRNA expressions of AR and ARV7 were detected in 22Rv1 cells transfected with RNF8 or RNF8C403S (*n* = 3). **H** AR and ARV7 protein expression levels in 22Rv1 cells transfected with RNF8 and RNF8C403S. Data of the qRT-PCR are presented as the ratio normalized to *RPS18* (means ± SEM). The statistical analysis is one-way ANOVA for (**A**, **C**); a two-sided unpaired *t*-test for (**D**, **E**, and **G**). *P < 0.05; **P < 0.01; ***P < 0.001; ****P < 0.0001. Numbers below the western blot result indicate the relative gray value normalized to the internal control (α-tubulin or β-actin).

To study whether RNF8 regulates *AR* transcription, RNF8 was knocked down in androgen-sensitive LNCaP, CRPC 22Rv1, and established enzalutamide-resistant LNCaP-EnzR cells by specific siRNA (Fig. 2A and Supplementary Fig. 3A). The efficacy of enzalutamide resistance of LNCaP-EnzR cells was determined by the CCK8 assay (Supplementary Fig. 2). RNF8 knockdown resulted in reduced expression of *AR* pre-mRNA in these PC cells detected by qRT-PCR with primers flanking *AR* gene exon 1 and intron 1 (Fig. 2B, C and Supplementary Fig. 3B). The mature mRNA levels of *AR* and *ARV7* also declined in RNF8 silenced cells (Fig. 2D and Supplementary Fig. 3C). The downregulation of AR and ARV7 protein levels was also observed in RNF8 silenced cells (Supplementary Fig. 4A, E). In contrast, overexpression of RNF8 increased the mRNA and protein levels of AR and ARV7 in PC cells (Fig. 2E, F and Supplementary Figs. 3D, 4B).

To detect whether the induction of *AR* transcription relied on the E3 ligase activity of RNF8, we examined that this effect of wild-type RNF8 was reduced by E3 ligase inactivation (RNF8C403S) (Fig. 2G, H and Supplementary Fig. 3E).

However, we observed no significant effects of RNF8 on AR and AR-Vs stability in 22Rv1 cells with protein synthesis inhibitor cycloheximide (CHX) treatment (Supplementary Fig. 4C, D). In addition, MG132 treatment could not reverse the reduction of AR and ARV7 expression by RNF8 depletion (Supplementary Fig. 4E).

These findings indicate that RNF8 promotes *AR* gene transcription via its E3 ligase activity and increases subsequent expression of AR and ARV7 in PC cell lines without affecting the protein stability.

### RNF8 interacts with c-Myc and modulates histone modifications at the c-Myc binding site in the *AR* gene

Several TFs have been identified to regulate *AR* gene transcription [44]. We searched for the potential interactors of RNF8 in PrePPI [45, 46] and investigated whether they act as AR’s TF. Among them, c-Myc was selected as a candidate [13, 44].

**Fig. 3:**
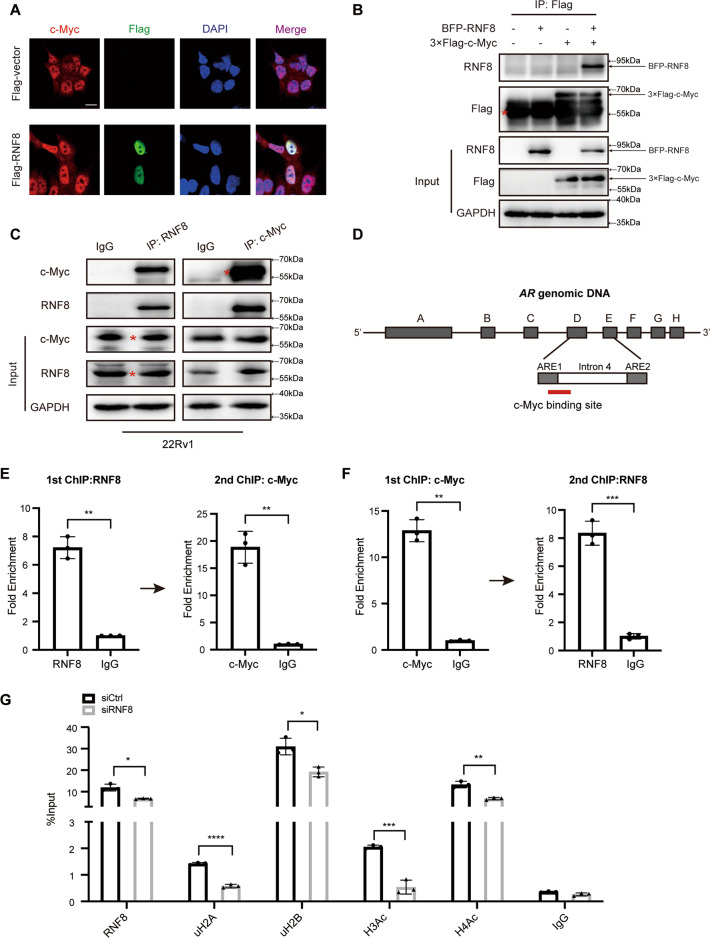
RNF8 interacts with c-Myc and modulates histone modifications at the c-Myc binding site in the *AR* gene. **A** Colocalization of Flag-RNF8 (green) and endogenous c-Myc (red) in 22Rv1 cells was detected by confocal. Scale bar, 10 µm. **B** The 22Rv1 cells were transfected with BFP-RNF8 and 3×Flag-c-Myc. The whole-cell lysates were performed immunoprecipitation by Flag M2 resin. BFP-RNF8 in the immunoprecipitated complex was detected by western blot using the RNF8 antibody. The asterisk indicates the heavy chain. **C** The 22Rv1 cell lysates were immunoprecipitated with RNF8 or c-Myc antibody followed by western blot. The asterisk indicates the specific bands. **D** Schematic representation of the c-Myc binding site in the *AR* gene. The letters A to H indicate the exons of the *AR* gene. **E**, **F** ChIP re-IP assay was utilized to examine RNF8 and c-Myc forming a complex on the c-Myc binding site of the *AR* gene. The precipitated chromatin was analyzed by the qRT-PCR using primers flanking the region in (**D**). **G** ChIP assay was performed in control and RNF8 silenced 22Rv1 cells by indicated antibodies. Data were presented as the fold enrichment relative to IgG control for ChIP re-IP (means ± SEM, *n* = 3); %input relative to the adjusted input for ChIP (means ± SEM, *n* = 3). *P < 0.05; **P < 0.01; ***P < 0.001; ****P < 0.0001 (two-sided unpaired *t*-test).

We confirmed the colocalization and association between RNF8 and c-Myc in 22Rv1 cells (Fig. 3A–C). However, RNF8 depletion did not influence *MYC* transcription in the PC cell lines (Supplementary Fig. 5A–C). It was reported that c-Myc binds to a 350-bp consensus site (c-Myc binding site) within the *AR* gene (Fig. 3D). This region is vital for inducing *AR* transcription [47, 48]. Consequently, the ChIP re-IP assay using RNF8 and c-Myc antibodies demonstrated that they were recruited together to this region (Fig. 3E, F).

As an E3 ligase, RNF8 classically ubiquitinates histone H2A and H2B [38]. The acetylation of histones H3 and H4 (H3Ac and H4Ac) was closely associated with epigenetic activation of transcription [49–52]. Here, the ChIP assay showed that uH2A/uH2B and H3Ac/H4Ac were inhibited at the c-Myc binding site within the *AR* gene when RNF8 was knockdown (Fig. 3G), implying crosstalk between RNF8-mediated ubiquitination of H2A/H2B and the acetylation of H3/H4.

These results indicate that RNF8 upregulates *AR* gene transcription by forming a complex with c-Myc to regulate histone modifications at the c-Myc binding site in the *AR* gene.

### RNF8 co-activates AR/ARV7-induced transactivation

**Fig. 4:**
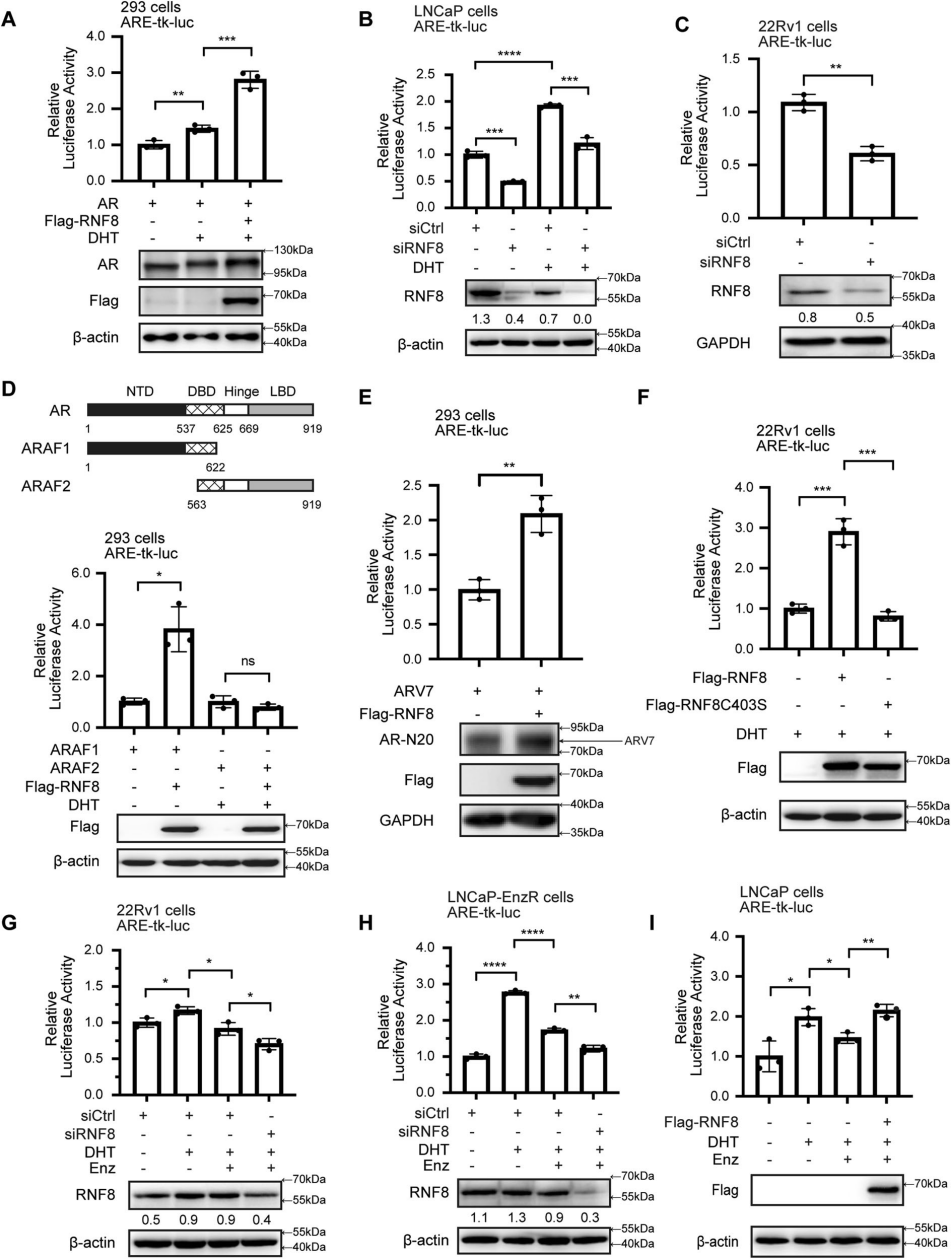
RNF8 co-activates AR/ARV7-induced transactivation. **A** HEK293 cells transfected with ARE-tk-luc, AR, vector, Flag-RNF8, and pRL-CMV treated with vehicle or DHT (10^−8^ M) for 24 h were measured luciferase activity. The expressions of AR and Flag-RNF8 were detected by western blot. **B** Luciferase activity was detected in the control and RNF8 silencing LNCaP cells with or without DHT treatment (10^−8^ M, 24 h). The efficacy of RNF8 silencing was examined by western blot. **C** RNF8 knockdown 22Rv1 cells and the control cells with ARE-tk-luc and pRL-CMV co-transfected were maintained in RPMI-1640 with 5% charcoal-stripped serum (CSS) for 24 h and then examined luciferase activity. RNF8 knockdown efficacy was determined by western blot. **D** Upper: schematic diagram of full-length AR and its mutations (ARAF1 and ARAF2). Numbers indicate amino acid positions; Lower: HEK293 cells were transfected with ARE-tk-luc, ARAF1, ARAF2, vector, Flag-RNF8, and pRL-CMV and measured luciferase activity. The expression of Flag-RNF8 was detected by western blot. **E** HEK293 cells were transfected with ARV7, ARE-tk-luc, vector, Flag-RNF8, and pRL-CMV. The cells were maintained in DMEM with 5% CSS for 24 h and examined luciferase activity. The expression of ARV7 and Flag-RNF8 were detected by western blot. **F** 22Rv1 cells were transfected with vector, Flag-RNF8, Flag-RNF8C403S, ARE-tk-luc, and pRL-CMV. The cells were treated with DHT (10^−8^ M) for 24 h. Then, luciferase activity was detected. Flag-RNF8 and Flag-RNF8C403S expressions were determined by western blot. **G**, **H** 22Rv1 and LNCaP-EnzR cells were transfected with control siRNA or siRNA targeting RNF8 for 24 h and then transfected with ARE-tk-luc and pRL-CMV for 24 h. The cells were treated with the vehicle, DHT (10^−8^ M), or enzalutamide (10 µM) for 24 h. Then luciferase activity was detected. RNF8 silencing was determined by western blot. **I** LNCaP cells were transfected with vector, Flag-RNF8, ARE-tk-luc, and pRL-CMV treated with the vehicle, DHT (10^−8^ M), or enzalutamide (10 µM). 24 h later, the cells were harvested for detecting luciferase activity. Flag-RNF8 expression was examined by western blot. Data were shown as mean relative LUC units ± SEM from three independent experiments. ns no significance, *P < 0.05, **P < 0.01, ***P < 0.001; ****P < 0.0001 (two-sided unpaired *t*-test). Numbers below the western blot resul*t* indicate the relative gray value normalized to the internal control (β-actin or GAPDH).

The Dual-luciferase reporter assay showed that overexpression of RNF8 induced the transactivation of AR in the presence of DHT (Fig. 4A). Luciferase activity of ARE driven by endogenous AR was reduced when RNF8 was silenced in LNCaP cells without or with DHT treatment (Fig. 4B). Knocking down of RNF8 also decreased endogenous AR transactivation in 22Rv1 cells in the absence of DHT (Fig. 4C), implying that RNF8 enhanced AR transactivation independent of androgen.

To investigate the effect of RNF8 on the transactivation-mediated by different transcription regions of AR (androgen-independent transcription region ARAF1 and androgen-dependent transcription region ARAF2) [53] (Fig. 4D upper), we separately detected the transactivation of ARAF1 and ARAF2. Compared to the control group, overexpression of RNF8 increased the transactivation of ARAF1, whereas it barely affected the transactivation of ARAF2 in the presence of DHT (Fig. 4D lower). This result indicated a potent effect of RNF8 on regulating the activity of the AR N-terminal. ARV7, which lacks the LBD domain in AR C-terminal, is involved in persistently transcription activation without androgen [54]. We found that RNF8 overexpression enhanced ARV7 transactivation in the absence of DHT (Fig. 4E).

Furthermore, we observed that the induction of endogenous AR transactivation by RNF8 overexpression was impaired by E3 ligase inactivation of RNF8 (RNF8C403S) (Fig. 4F). This result suggested that RNF8 promoted AR transactivation in an E3 ligase-dependent manner. Moreover, silencing RNF8 further attenuated AR transactivation in enzalutamide-resistant 22Rv1 and LNCaP-EnzR cells with enzalutamide treatment (Fig. 4G, H). On the contrary, overexpression of RNF8 reversed the reduction of AR transactivation by enzalutamide in LNCaP cells (Fig. 4I).

These results suggest that RNF8 induces AR/ARV7 transactivation androgen independently. Inducing AR transactivation by RNF8 relies on its E3 ligase activity. These functions of RNF8 confer PC cell’s resistance to the inhibitory effect of enzalutamide on AR/ARV7 transactivation.

### RNF8 upregulates the transcription of endogenous AR target genes in prostate cancer cells

**Fig. 5:**
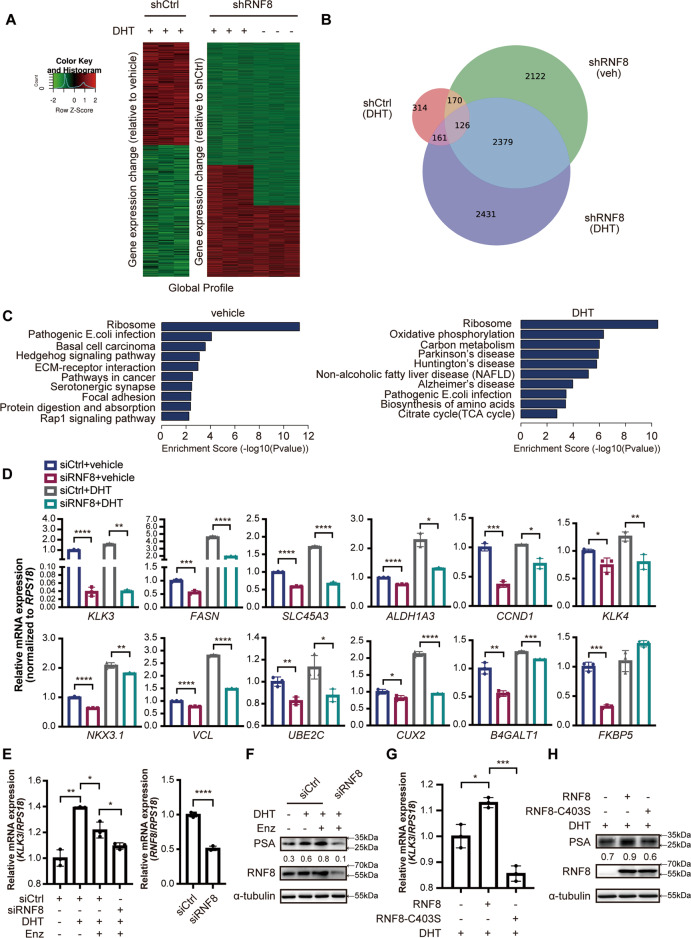
RNF8 upregulates the transcription of endogenous AR target genes in prostate cancer cells. **A** mRNA-seq analysis profile of control and RNF8 silencing 22Rv1 cells treated without or with DHT (10^−8^ M) for 24 h. The heat map displays gene expression change of DHT-induced genes upon RNF8 knockdown. Fold change is indicated as left. **B** Venn diagram showed DHT-regulated genes in shCtrl and shRNF8 cells in the absence or presence of DHT (10^−8^ M). **C** Biological processes of DHT-induced transcripts impacted by RNF8 knockdown without or with DHT (10^−8^ M) treatment as revealed by KEGG pathway analysis. **D** The transcription levels of indicated genes in control or RNF8 silencing 22Rv1 cells treated without or with DHT (10^−8^ M) for 24 h (*n* = 3). **E**, **F** The mRNA and protein levels of KLK3 (PSA) in control or RNF8 silencing 22Rv1 cells with the vehicle, DHT (10^−8^ M) or enzalutamide (10 µM) treatment for 24 h. **G**, **H** The mRNA and protein levels of KLK3 (PSA) in 22Rv1 cells transfected with Flag-RNF8 or Flag-RNF8C403S treated with DHT (10^−8^ M) for 24 h (*n* = 3). Data of the qRT-PCR were presented as the ratio normalized to *RPS18* (means ± SEM). *P < 0.05; **P < 0.01; ***P < 0.001; ****P< 0.0001 (two-sided unpaired *t*-test). Numbers below the western blot result indicate the relative gray value normalized to the internal control (α-tubulin).

To examine whether RNF8 regulated AR-mediated genes transcription, we performed transcriptome profiling by mRNA-sequencing in control and RNF8 knockdown 22Rv1 cells treated without or with DHT (SRP074464, GSE149581) [18]. The analysis showed that 771 differentially expressed genes (DEGs) were partially regulated by RNF8 silencing in the absence or presence of DHT (Fig. 5A and Supplementary Table 4), suggesting a role of RNF8 in regulating global AR target genes transcription. The Venn diagram exhibited that knockdown of RNF8 altered the expression of 296 and 287 DEGs without and with DHT treatment, respectively (Fig. 5B). Among these genes, RNF8 silencing modulated 126 DEGs in both the absence and presence of DHT, implying that RNF8 participated in AR- and AR-Vs-mediated transcription regulation. The enrichment analysis of the KEGG pathway showed that these DEGs were involved in several inner- or inter-cellular processes and human diseases in the absence or presence of DHT (Fig. 5C).

Subsequently, we examine the transcription of a subset of AR target genes in RNF8 silencing 22Rv1 cells without or with DHT treatment by the qRT-PCR. The transcription of *KLK3* (*PSA*), *FASN*, *SLC45A3*, *ALDH1A3*, *CCND1*, *KLK4*, *NKX3.1*, *VCL*, *UBE2C*, *CUX2*, and *B4GALT1* decreased when RNF8 was knockdown in both the absence and presence of DHT (Fig. 5D, Supplementary Fig. 6A), while the transcription of *FKBP5*, *TACC2*, *GTSE1*, *CCNA2*, *ABHD2*, *KRT18*, and *TMPRSS2* declined in RNF8 silencing cells without or with DHT treatment (Fig. 5D and Supplementary Fig. 6A–C), confirming the role of RNF8 in regulating AR- and AR-Vs-mediated transcription. Some of these genes expression was positively correlated with the expression of RNF8 in PC samples analyzed by GEPIA (Supplementary Table 5). The siRNA-mediated RNF8 knockdown efficacy was also determined (Supplementary Fig. 6B, C).

RNF8 silencing further decreased the mRNA and protein levels of KLK3 (PSA) in 22Rv1 cells treated with enzalutamide (Fig. 5E, F and Supplementary Fig. 6D). In addition, ectopic expression of RNF8C403S compromised the induction effect of RNF8 on KLK3 (PSA) expression (Fig. 5G, H and Supplementary Fig. 6E), indicating that RNF8 upregulated AR targets relying on its E3 ligase activity.

The above results suggest that RNF8 globally activates AR/AR-Vs-mediated transcription activity in an E3 ligase-dependent manner.

### RNF8 promotes the recruitment of AR/ARV7 to the *KLK3* promoter

**Fig. 6:**
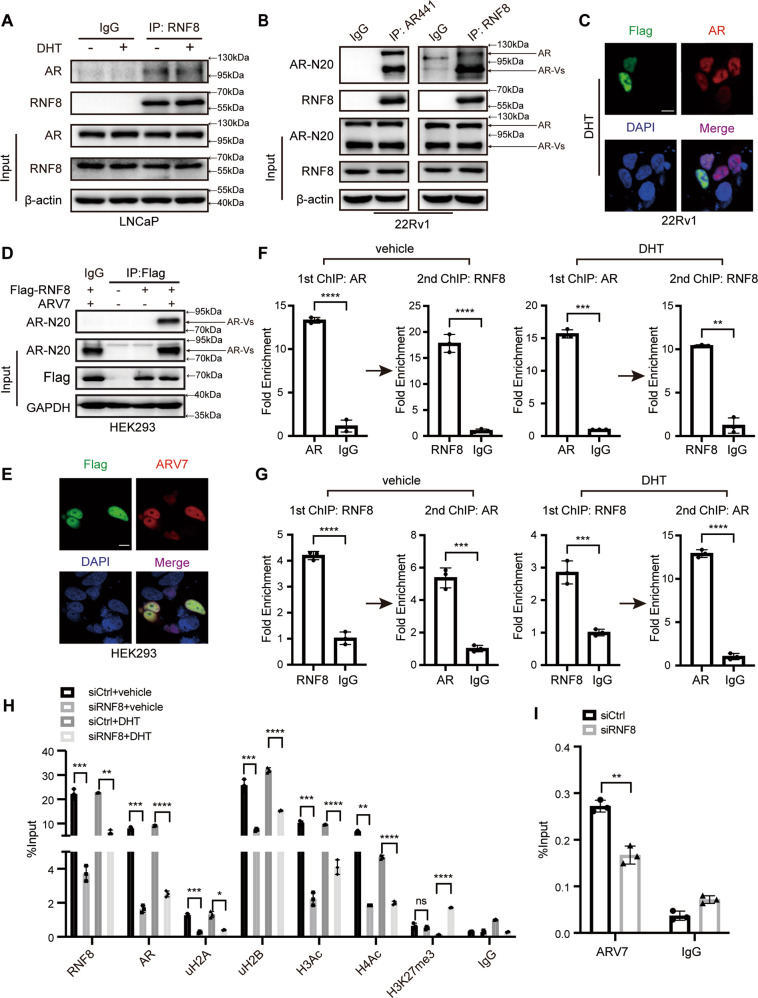
RNF8 promotes the recruitment of AR/ARV7 to the *KLK3* promoter. **A** The lysates from LNCaP cells treated without or with DHT (10^−8^ M) for 24 h were immunoprecipitated by RNF8 or IgG antibody. Precipitated proteins were detected by western blot. **B** 22Rv1 cells were maintained in RPMI-1640 with 5% CSS for 24 h, and the cell lysates were immunoprecipitated by indicated antibodies. Precipitated proteins were detected by western blot. **C** 22Rv1 cells were co-expressed with AR and Flag-RNF8 and treated with DHT (10^−8^ M) for 24 h. Then, the cells were stained with indicated antibodies. Scale bar, 10 µm. **D** HEK293 cells with Flag-RNF8 and ARV7 co-transfected were cultured in DMEM with 5% CSS for 24 h, and the cell lysates were immunoprecipitated by anti-Flag M2 resin and IgG. The precipitates were immunoblotted by indicated antibodies. **E** HEK293 cells were co-transfected with Flag-RNF8 and ARV7 and were maintained in RPMI-1640 with 5% CSS for 24 h. Then, the cells were stained with indicated antibodies. Scale bar, 10 µm. **F**, **G** ChIP re-IP was performed in 22Rv1 cells treated without or with DHT (10^−8^ M) for 24 h using RNF8/AR antibody and IgG as a negative control. The precipitated chromatin was analyzed by the qRT-PCR using primers flanking the *KLK3* (*PSA*) AREI/II. **H**, **I** ChIP assays were performed in control or RNF8 knockdown 22Rv1 cells treated without or with DHT (10^−8^ M) for 24 h by indicated antibodies Data were presented as the fold enrichment relative to IgG control for ChIP re-IP (means ± SEM, *n* = 3); %input relative to the adjusted input for ChIP (means ± SEM, *n* = 3). *P < 0.05; **P < 0.01; ***P < 0.001; ****P < 0.0001; ns no significance (two-sided unpaired *t*-test).

Surprisingly, we detected that RNF8 interacted with AR in LNCaP cells both with and without DHT (Fig. 6A). We also observed the interaction and colocalization of RNF8 and AR/AR-Vs in 22Rv1 cells without DHT treatment (Fig. 6B, C). Furthermore, RNF8 colocalized and interacted with ARV7 (Fig. 6D, E).

The ChIP re-IP assay, performed in 22Rv1 cells treated without or with DHT by RNF8 and AR antibodies, showed that RNF8 and AR both appeared at the ARE of the *KLK3* (*PSA*) promoter (*KLK3*-AREI/II) (Fig. 6F, G), confirming that they formed a complex at this region. In the ChIP assays targeting *KLK3*-AREI/II in control and RNF8 knockdown cells in the absence or presence of DHT, both AR and ARV7 recruitment was reduced by RNF8 silencing (Fig. 6H, I). The uH2A/uH2B and H3Ac/H4Ac decreased in RNF8 knockdown cells (Fig. 6H). However, the transcriptionally repressive tri-methylation level of H3K27 (H3K27me3) [55] increased in RNF8 silencing cells with DHT treatment (Fig. 6H).

These results imply that RNF8 promotes AR/ARV7 recruitment at the ARE, where RNF8-dependent ubiquitination of H2A/H2B may cause transcriptionally active histone modifications to increase but repressive ones to decrease.

### RNF8 depletion inhibits cell proliferation and enhances enzalutamide sensitivity in prostate cancer cell lines

**Fig. 7:**
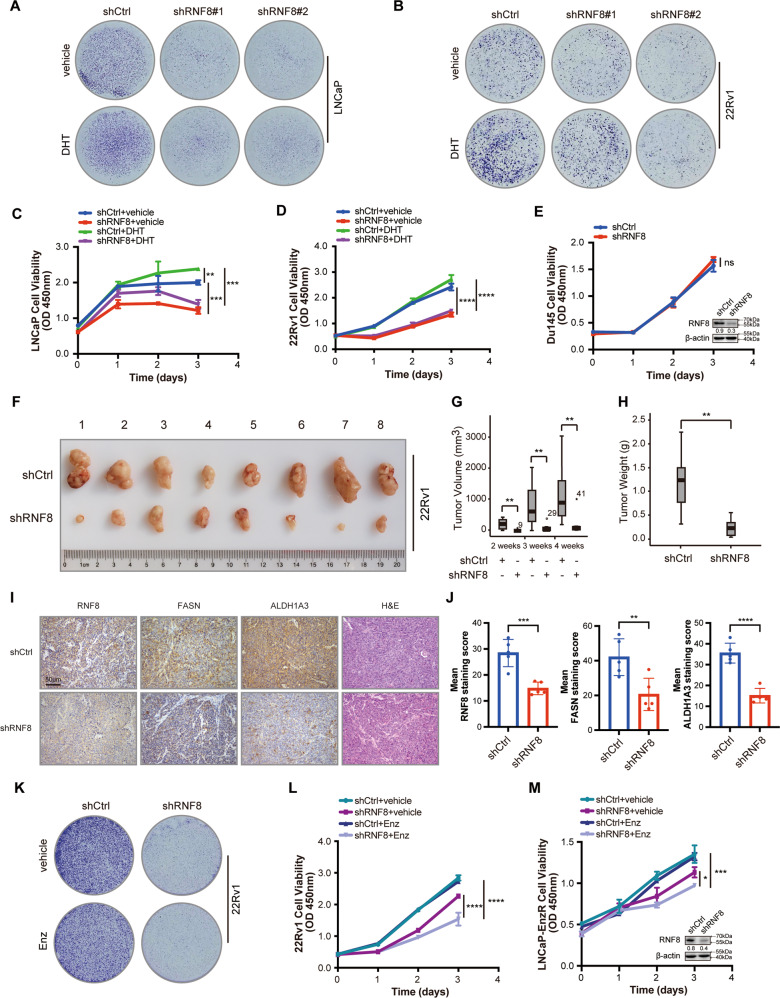
RNF8 depletion inhibits cell proliferation and enhances enzalutamide sensitivity in prostate cancer cell lines. **A**, **B** Control and stable RNF8 knockdown LNCaP (**A**) and 22Rv1 (**B**) cells were treated with vehicle or DHT (10^−8^ M) for 10 days. The cell colonies were stained with Coomassie blue. **C**, **D** Control and stable RNF8 knockdown LNCaP (**C**) and 22Rv1 (**D**) cells were treated with vehicle or DHT (10^−8^ M) for 0, 1, 2, 3 days. The cell viability was detected by the CCK8 proliferation assay. Data were means ± SEM (*n* = 3). **P < 0.01; ***P < 0.001; **** *P* < 0.0001 (two-sided unpaired *t*-test). **E** The proliferation of control and stable RNF8 knockdown DU145 cells was examined by the CCK8 assay. Data were means ± SEM (*n* = 3). ns no significance (two-sided unpaired *t*-test). RNF8 knockdown efficacy was determined by western blot. Numbers under the western blot bands indicate the relative gray value normalized to β-actin. **F** The photograph for the xenografts from NOD/SCID mice injected subcutaneously with control and stable RNF8 knockdown 22Rv1 cells (*n* = 8). **G**, **H** The median of tumor volume (**G**) and tumor weight (**H**) from (**F**). **P < 0.01 (two-sided Mann–Whitney test). **I** Representative images of xenograft tumor tissues in (**F**) staining with RNF8, FASN, and ALDH1A3. The H&E staining was also presented. Scale bar, 50 μm. **J** Statistical analysis of the mean staining intensity of RNF8, FASN, and ALDH1A3 in xenograft tumor tissues in (**I**). **P < 0.01; ***P < 0.001; ****P < 0.0001 (two-sided unpaired *t*-test). **K** Control and stable RNF8 knockdown 22Rv1 cells were treated with vehicle or enzalutamide (10 µM) for 10 days. The cell colonies were stained with Coomassie blue. **L**, **M** Control and stable RNF8 knockdown 22Rv1 (**L**) and LNCaP-EnzR (**M**) cells were treated with vehicle or enzalutamide (10 µM) for 0, 1, 2, 3 days. The cell viability was detected by the CCK8 assay. Data were means ± SEM (*n* = 3). *P < 0.05; ***P < 0.001; ****P < 0.0001 (two-sided unpaired *t*-test).

AR signal acts as the critical driver of PC and CRPC development. Thus, we investigate whether RNF8 is essential for AR-dependent PC and CRPC growth. We examined that RNF8 silencing inhibited the growth of LNCaP and 22Rv1 cells both without and with DHT treatment (Fig. 7A, B), which was confirmed by the growth curve analyses in control and RNF8 knockdown LNCaP and 22Rv1 cells (Fig. 7C, D). The efficacy of RNF8 knockdown in LNCaP and 22Rv1 cells was determined by western blot (Supplementary Fig. 7A, B). However, RNF8 depletion might only affect the proliferation of AR-positive PC cells, as there was no significant change in AR-negative DU145 cells growth with RNF8 silencing (Fig. 7E).

Subsequently, we injected stable RNF8 knockdown or control 22Rv1 cells subcutaneously into male NOD/SCID mice and measured the tumor volume every week. The size of tumors grown from control cells was bigger in contrast to that from RNF8 knockdown cells (Fig. 7F). The volume increasing rate and the weight of control cells exhibited greater than that of RNF8 knockdown cells (Fig. 7G, H). Furthermore, the expression of AR target genes FASN and ALDH1A3 declined in RNF8 silenced tumors (Fig. 7I, J and Supplementary Figs. 1C, 7C).

Moreover, compared to the control group, RNF8 knockdown further reduced the proliferation of enzalutamide-resistant 22Rv1 and LNCaP-EnzR cells with enzalutamide treatment (Fig. 7K–M), indicating that RNF8 depletion enhanced enzalutamide sensitivity in CRPC cell lines.

Generally, these results suggest that RNF8 is required for cell growth and enzalutamide resistance in advanced PC progression.

## Discussions

**Fig. 8:**
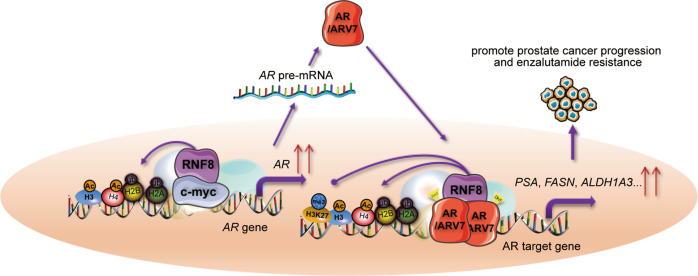
Schematic representation of RNF8 upregulating AR/ARV7 action. RNF8 forms a complex with c-Myc being recruited together to the c-Myc binding site in the *AR* gene, inducing the ubiquitination of H2A/H2B and acetylation of H3/H4. This effect causes increased transcription of the *AR* pre-mRNA, resulting in enhanced AR/ARV7 expression. RNF8 interacts with AR/ARV7 binding onto the ARE of AR target gene, promoting H2A/H2B ubiquitination and H3/H4 acetylation while attenuating H3K27 tri-methylation around this region. This effect ultimately leads to increased expression of AR target genes, which may accelerate advanced PC progression and enzalutamide resistance.

Our study identifies RNF8 as an activator of AR/ARV7, which undergoes an unexpected mechanism participating in advanced PC progression and enzalutamide resistance with important clinical implications. Our data demonstrate that RNF8 upregulates *AR* gene transcription by forming a complex with c-Myc, increasing the ubiquitination of H2A/H2B and acetylation of H3/H4 at the c-Myc binding site in the *AR* gene. Furthermore, RNF8 interacts with AR/ARV7 to be recruited together to the ARE and regulates histone modifications around this region. RNF8 co-activates the transcription of AR target genes, which may be responsible for the progression of advanced PC and enzalutamide resistance (Fig. 8).

The bioinformatic analysis suggests that the high expression of *RNF8* is positively associated with poor survival of PC patients (Fig. 1A, B). In line with this, we find that there was a significant trend of higher RNF8 expression with higher GS in human PC tissues (Fig. 1C, D and Supplementary Table 2). RNF8 expression is high in AR-positive PC and CRPC cells (Fig. 1E), consistent with bioinformatics analyses in PC (Fig. 1F, G). Furthermore, *RNF8* mRNA expression is positively correlated with *AR* in PC (Fig. 1H). These findings indicate a significant role of RNF8 in advanced PC progression and the positive relationship with AR in PC.

Corresponding to our hypothesis, silencing RNF8 causes a reduction of *AR* pre-mRNA expression, and subsequent AR*/*ARV7 mRNA and protein levels in hormone-sensitive, CRPC, and enzalutamide-resistant PC cells (Fig. 2A–D and Supplementary Figs. 3A–C, 4A, E). On the contrary, overexpression of RNF8 increases the mRNA and protein levels of AR/ARV7 in PC cells (Fig. 2E, F and Supplementary Figs. 3D, 4B). Since *AR* and *ARV7* mature mRNA are both derived from the same *AR* pre-mRNA, the alterations of AR and ARV7 expressions by RNF8 silencing or overexpression primarily result from the changes in *AR* pre-mRNA expression. Notably, the E3 ligase activity of RNF8 is required for its function in the regulation of *AR* transcription (Fig. 2G, H and Supplementary Fig. 3E). Although RNF8 participates in protein ubiquitination and degradation [31, 56], we find no significant changes in protein stability of AR/ARV7 by RNF8 overexpression or knockdown (Supplementary Fig. 4C–E). These results suggest the upregulation function of RNF8 on *AR* gene transcription in an E3 ligase-dependent manner.

Concerning how RNF8 upregulates *AR* transcription, we search for an interactor of RNF8 which is a TF of AR. As a predicted interactor of RNF8, c-Myc acting as an influential TF of AR [13, 44] has been selected for further study. Although we have reported in our previous study that RNF8 is an activator for *MYC* transcription via regulating β-catenin nuclear translocation in WNT/APC constructively activated colon cancer (CCR) cells [39], c-Myc mRNA and protein levels are not changed substantially in RNF8 silencing PC cells (Supplementary Fig. 5A–C), which may be due to the different activation extent of WNT signaling between CCR and PC cells. We provide evidence that RNF8 interacts with c-Myc and forms a complex at the c-Myc binding site within the *AR* gene (Fig. 3A–F).

Furthermore, RNF8 increases the ubiquitination of H2A/H2B and the acetylation of H3/H4 around the c-Myc binding site in the *AR* gene (Fig. 3G). Moreover, we identify the association between RNF8 and AR/ARV7, forming a complex at the ARE (Fig. 6A–G). The depletion of RNF8 inhibits the recruitment of AR/ARV7 at the ARE in 22Rv1 cells (Fig. 6H, I), which may be caused by the decrease of AR/ARV7 expression in RNF8 silencing cells (Fig. 2D and Supplementary Figs. 3C, 4A, E). Moreover, H2A/H2B ubiquitination and H3/H4 acetylation decreased, while H3K27 tri-methylation increased around the ARE in RNF8 knockdown cells (Fig. 6H). H2A and H2B are well-known targets for RNF8-mediated ubiquitination [57, 58]. Although the uH2A at K119 is linked to polycomb-repressive complex (PRC) mediated gene silencing, uH2A also provides a signal for ZRF1 recruitment, which displaces PRC1 from chromatin to activate transcription [59, 60], suggesting a possible role of uH2A in transcriptional activation via recruitment of other proteins. The uH2B at K120 is a noted histone modification for transcription activation by offering a signal hub for downstream histone modification or direct decompacting of high-order chromatin structure [60, 61]. Thus, in PC cells, RNF8-induced uH2A/uH2B may ultimately cause “trans-histone” crosstalk with other histone modifications around the c-Myc binding site in the *AR* gene and the ARE, upregulating AR/ARV7 action in an E3 ligase-dependent manner (Figs. 4, 5).

Active AR signaling is essential for the proliferation of PC [2]. We observe that silencing RNF8 inhibits the growth of AR-positive LNCaP and 22Rv1 cells both without and with DHT treatment (Fig. 7A–D). However, knockdown RNF8 has barely any effect on AR-negative DU145 cells growth (Fig. 7E). These results suggest that RNF8 promotes PC growth by targeting AR signaling. Moreover, RNF8 knockdown retards the proliferation of CRPC xenograft tumors by reducing the expression of AR target genes FASN and ALDH1A3 (Fig. 7F–J).

The enzalutamide is a potent AR inhibitor, approved by FDA for treating CRPC [4]. Unfortunately, drug resistance ultimately develops with mechanisms including AR amplification, AR mutation, and AR splice variants [5, 6]. As discussed above, RNF8 increases AR/ARV7 expression in CRPC and enzalutamide-resistant PC cells (Fig. 2D–F and Supplementary Figs. 3C, D, 4A–E). Silencing RNF8 further reduces AR/ARV7 transactivation and PSA expression in enzalutamide-resistant PC cells treated with enzalutamide (Figs. 4G, H, 5E, F and Supplementary Fig. 6D). Overexpression of RNF8 impairs the inhibitory effect of enzalutamide on AR transactivation in androgen-dependent PC cells (Fig. 4I). Subsequently, RNF8 knockdown sensitizes enzalutamide-resistant PC cells 22Rv1 and LNCaP-EnzR to enzalutamide treatment (Fig. 7K–M). These results imply that RNF8 may confer PC cell’s resistance to ADT via activating AR/ARV7, indicating that RNF8 may be an effective target for ameliorating the enzalutamide resistance.

Collectively, the modulation effect of RNF8 on AR/ARV7 action in our work unravels novel molecular and pathological functions of RNF8 in advanced PC progression and enzalutamide resistance. Our data provide new insights into understanding the progression of advanced PC, raising RNF8 as a candidate for developing a therapy to treat advanced PC and overcome enzalutamide resistance.

## Materials and methods

### Bioinformatics analysis

Overall survival and disease-free survival data of *RNF8* mRNA low expression or high expression patients and the correlation of *RNF8* and *AR* mRNA expression were analyzed by the GEPIA database [41].

*RNF8* mRNA expression and clinicopathological characteristics of PC patients were downloaded from TCGA, preprocessed, and integrated by R. The evaluation of the effect of clinicopathological characteristics and *RNF8* mRNA expression on the OS of PC patients was analyzed in SPSS using the ROC curve, UV COX, and MV COX regression. The trend of *RNF8* mRNA expression with GS was analyzed by the Jonckheere–Terpstra test in SPSS.

Transcriptome data of NCBI GEO datasets of PC were downloaded, preprocessed, and integrated. Groups were separated as AR-positive and AR-negative PC. The mean expression for RNF8 in both groups was calculated and compared.

Datasets of primary PC and CRPC tissues (GSE74367) [43] were downloaded from NCBI Gene Expression Omnibus (GEO) databases and analyzed by using RNF8 and AR signatures.

### Plasmids construction

Flag-RNF8 and Flag-RNF8C403S were reconstructed from His-RNF8 friendly donated by Yukihiko Takano [33]. AR and ARE-tk-luc plasmids were constructed as described previously [62, 63]. The pcDNA-ARV7 plasmid was a gift from Dr. Luo [64].

### Cell culture

LNCaP, CWR22Rv1 (22Rv1), PC3, DU145, and HEK293 cells were gifts from the Key Laboratory of Cell Biology of China Medical University. VCaP cells were obtained from the Chinese Academy of Sciences. These cell lines were authenticated by STR profiling. LNCaP, 22Rv1, and DU145 cells were routinely maintained in RPMI-1640. VCaP and HEK293 cells were cultured in DMEM and PC3 in Ham’s F12K. LNCaP-EnzR cells were maintained in RPMI-1640 plus enzalutamide (10 μM). Media were supplemented with 10% fetal bovine serum (FBS) and 10 g/L streptomycin and penicillin.

### The siRNA transfection

The siRNA against RNF8 and a control siRNA (designed and synthesized by Damaracon) were transfected in LNCaP, LNCaP-EnzR, and 22Rv1 cells using Lipofectamine 3000 transfection reagent (Thermo Fisher) following the manufacturer’s instructions. Followed by incubation for 48 h to allow degradation of targeted mRNA, and the cells were harvested for the qRT-PCR, western blot, ChIP assays, and luciferase assay (Promega). The siRNA sequences targeting RNF8 were shown in Supplementary Table 7.

### Lentiviral production and infection

RNF8 shRNA and control shRNA lentivirus were purchased from Shanghai GENECHEM Company. Stable RNF8 silencing and control cells were selected with puromycin (2 μg/ml) after infection. The shRNA sequences against RNF8 were shown in Supplementary Table 8.

### Immunohistochemical analysis of prostate cancer tissues

The prostate/PC tissue specimens from clinical prostatectomy specimens in the First Hospital and the Second Hospital Affiliated to China Medical University and 463 Military Hospital and the xenograft tumor sections were formalin-fixed and paraffin-embedded. Multicentre ethical approval for data collection and tissue utility was granted by the Human Research Ethics Committee of the above hospitals. In these tissue sections, anti-RNF8 (ab65739 Abcam), anti-FASN (10624-2-AP Proteintech), anti-ALDH1A3 (25167-1-AP Proteintech), goat anti-rabbit/mouse IgG (AS070, AS071 ABclonal), and avidin-biotin-conjugated second antibodies (Vectastain ABC Elite kit; Vector Laboratories) were used to detect indicated proteins expression levels. The signals were visualized with diaminobenzidine, and the nuclei were counterstained with hematoxylin. The slides were also stained with hematoxylin-eosin (H&E).

For clinical tissue, the RNF8 staining was assessed by double-blinded evaluation by two observers in an intra-observer way [65]. The nuclear staining intensity of RNF8 was scored as 0–3 (0 for negative; 1 for light brown; 2 for brown; 3 for dark brown). The staining score was used to assess the level of staining. Staining score is the sum of intensity score multiple by the percentage of positive staining cells. The intra-observer variability is assessed by the interclass correlation coefficient (ICCC) of RNF8 staining evaluation by two observers, which is summarized in Supplementary Table 9. The trend of RNF8 staining score with GS in PC samples was analyzed by the Jonckheere–Terpstra test.

For the xenograft tumor section, the staining intensity (IOD value) of RNF8, FASN, or ALDH1A3 was assessed by Image-Pro Plus 6.0.

### Antibodies, immunoprecipitation, and immunofluorescence

The first antibodies used in this study are anti-RNF8 (ab65739 Abcam, #09-813 Millipore, and sc-271462 Santa Cruz Biotechnology), anti-AR-N20 (22089-1-AP Proteintech), anti-AR-441 (MA5-13426 Thermo scientific), anti-ARV7 (31-1109-00 RevMab Biosciences), anti-c-Myc (10828-1-AP Proteintech, ab32072 Abcam), anti-Flag (M2, F3165 Sigma and A00187 Genescript), anti-GAPDH (#KC5G4 Shanghai Kangchen), anti-α-tubulin (AC012 ABclonal), anti-uH2A-K119 (#05-678 Millipore), anti-uH2B-K120 (#5546 Cell Signaling Technology), anti-H3Ac (06-599 Upstate Biotechnology) anti-H4Ac (06-866 Upstate Biotechnology), anti-H3K27me3 (ab6002 Abcam), anti-PSA (10679-1-AP Proteintech), and anti-β-actin (AC004 ABclonal).

In the immunoprecipitation experiment, for 22Rv1 and LNCaP cells, the whole-cell extracts were prepared using TNE buffer (10 mM Tris-HCl, pH 7.5; 1% NP-40; 0.15 M NaCl; 1 mM EDTA, pH8.0) with 1×protease inhibitor cocktail (Roche Molecular Biochemicals). For 22Rv1 and HEK293 cells, BFP-RNF8/3×Flag-c-Myc or Flag-RNF8/pcDNA-ARV7 expression plasmids were transiently transfected using the jetPRIME reagent (Polyplus) or HiGene transfection agent (Applygen). The equal protein amounts were immunoprecipitated with anti-RNF8, anti-AR (441), anti-c-Myc antibody, or anti-Flag M2 resin. The immunoprecipitated protein complexes were washed three times with TNE buffer supplemented with the protease inhibitor cocktail. The whole-cell lysates and immune complexes were detected by western blot using indicated primary and secondary antibodies. For endogenous immunoprecipitation of RNF8 and c-Myc/AR, light-chain-specific secondary antibody (ab99697 Abcam, A25012 IPKine) was used. And chemiluminescence detection was performed according to the manufacturer’s instructions (GE-Healthcare). The protein concentration of the whole-cell extracts was determined by a standard Bradford assay.

In the immunofluorescence experiment, HEK293 cells were transfected with Flag-RNF8 together with AR or ARV7 expression plasmids. Four to six hours after transfection, the culture media were replaced with fresh indicated media with 5% charcoal-stripped serum (CSS) and supplemented with vehicle (ethanol) or DHT (10^−8^ M). 22Rv1 cells were transfected with Flag-RNF8 for 4–6 h and changed with fresh media. Twenty-four hours later, the cells were divided into 12-well culture plates with polylysine-coated slides. HEK293 cells were maintained in media containing 5% CSS and supplemented with vehicle (ethanol) or DHT (10^−8^ M), while 22Rv1 cells were cultured in normal media for 24 h. The cells were fixed with 4% paraformaldehyde and permeabilized with 0.1% TritonX-100. HEK293 cells and 22Rv1 cells were incubated with anti-AR (AR-N20), anti-Flag, or anti-c-Myc antibodies. Incubation was followed by washing in 1×PBS, and the cells were then incubated with relevant secondary FITC-/Alexa Fluor 488, or Cy5/Alexa Fluor 594-conjugated antibodies (Jackson Immunoresearch Laboraories Inc and Thermo Fisher). The nuclei were stained with DAPI (Roche). Immunofluorescence staining was visualized using Zeiss confocal laser scanning system 510.

### Chromatin immunoprecipitation

RNF8 knockdown and control 22Rv1 cells were cultured in RPMI-1640 containing 5% CSS and with ethanol or DHT (10^−8^ M) for 24 h. The cells were cross-linked with 1% formaldehyde in a 37 °C incubator for 10 min. Then, the cells were resuspended in SDS lysis buffer (1% SDS; 10 mM EDTA; 50 mM Tris-HCl, pH8.1) with a protease inhibitor cocktail (Roche Molecular Biochemicals) and sonicated six times for 10 s at 60% attitude (Handy Sonic, Model UR-20P) followed by centrifugation at 12,000 rpm at 4 °C for 10 min. Supernatants were diluted in ChIP dilution buffer (0.01% SDS; 1.1% TritonX-100; 1.2 mM EDTA; 16.7 mM Tris-HCl, pH8.1) followed by immunoclearing with the Protein A Agarose/Salmon Sperm DNA (Millipore) for 30 min at 4 °C. The immunoprecipitates were incubated with specific antibodies overnight at 4 °C. Then, the Protein A Agarose/Salmon Sperm DNA was added, and the protein–DNA complexes were washed sequentially with low-salt buffer, high-salt buffer, LiCl buffer, and TE buffer. The protein–DNA complexes were eluted and reversed crosslinking at 65 °C overnight. DNA fragments were purified by phenol/chloroform and absolute ethyl alcohol and then were analyzed by the qRT-PCR. The primer sequences of *KLK3*-AREI/II were as follows: forward 5′-GCCAAGACATCTATTTCAGGAGC-3′, and reverse 5′-CCCACACCCAGAGCTGTGGAAGG-3′; the primer sequences of the c-Myc binding site in the *AR* gene were as described previously [48].

### ChIP re-IP

The ChIP aspects were performed for 22Rv1 cells treated without or with DHT (10^−8^ M) as described above. The complexes were eluted from the primary IP by incubation with 10 mM DTT at 37 °C for 30 min and diluted in Re-ChIP buffer (1% TritonX-100; 2 mM EDTA; 150 mM NaCl; 20 mM Tris-HCl, pH8.1) with 1×protease inhibitor cocktail (Roche Molecular Biochemicals) followed by re-IP with second antibodies. DNA was analyzed by the qRT-PCR using *KLK3* promoter primers as described in the ChIP experiment.

### Transfection and luciferase assay

Before transfection, LNCaP, LNCaP-EnzR, 22Rv1, and HEK293 cells were grown in indicated media with 5% CSS. Cells were transfected with indicated plasmids/siRNA, pRL-CMV, and the reporter gene carrying ARE using jetPRIME reagent (Polyplus). Four to six hours later, the media was changed for fresh ones with 5% CSS and vehicle (ethanol) or DHT (10^−8^ M). After 24 h, cells were conducted to detect luciferase activities by the Dual-Luciferase Reporter Assay System (Promega) as the manufacturer described.

### RNA-seq analysis, differentially expressed genes, and bioinformatics analysis

Total RNA was extracted from stable RNF8 knockdown 22Rv1 cells pretreated with ethanol or DHT (10^−8^ M) for 24 h. Each experiment was repeated three times. RNA quality and quantity were evaluated by NanoDrop ND-2000 (Thermo Fisher Scientific, Waltham, MA, USA). RNA integrity was analyzed by non-denaturing agarose gel electrophoresis. The quality library of RNA was detected by Agilent 2100 Bioanalyzer. After removing the rRNAs from 1 μg total RNA, libraries were constructed using TruSeq Stranded Total RNA Library Prep Kit (Illumina, San Diego, CA, USA). The library quality was controlled by the BioAnalyzer 2100 system (Agilent Technologies, Inc., USA). About 10 pM libraries were sequenced for 150 cycles on Illumina HiSeq Sequencer according to the manufacturer’s instructions. Paired-end reads were harvested from Illumina HiSeq 4000 sequencer and were quality controlled by Q30. After 3′ adapter-trimming and the low-quality reads removed by cut-adapt software, the high-quality clean reads were aligned to the reference genome (UCSC hg19) with hisat2 software. Guiding by the Ensembl gtf gene annotation file, the gene-level FPKM getting by cuffdiff software (part of cufflinks) was used as mRNA expression profiles, and fold change and *p* value were calculated based on FPKM by *t*-test, differentially expressed mRNA were identified. Genes with fold change ≥2.0 and *p* value ≤0.05 between two populations were kept in the heat map. GO (Gene Ontology) and Pathway enrichment analysis was performed based on the differentially expressed mRNAs. All the above analysis was performed by Cloud-Seq Biotech (Shanghai, China).

### Accession number

The mRNA sequencing data have been deposited in the NCBI GEO database with the accession GSE149581.

### RNA isolation, reverse transcription, and qRT-PCR

Total RNA was isolated using RNAiso plus (TAKARA). According to the manufacturer’s instruction, reverse transcription was performed using PrimeScript^™^ RT reagent kit with gDNA Eraser (Perfect Real Time) (TAKARA). The cDNAs were quantified by the qRT-PCR using TB Green^®^*Premix Ex Taq*^™^ II (Tli RNaseH Plus) (TAKARA) on LightCycler 96 instrument (Roche). Primers used to detect mRNA expression were designed by Sangon Biotech (the sequence of the primers was shown in Supplementary Table 6). Gene expression levels were calculated relative to the housekeeping genes *RPS18*, *PPIA*, and *ACTB*. The *PPIA* and *ACTB* primer pairs are finished products purchased from Sangon Biotech.

### In vitro cell growth assay and cell proliferation assay

For in vitro cell growth assay, 2 × 10^4^ stable RNF8 knockdown or control LNCaP and 22Rv1 cells were plated in 35-mm dishes and maintained in RPMI-1640 with 5% CSS supplemented with ethanol or DHT (10^−8^ M) for 10 days or cultured in RPMI-1640 with 10% FBS plus DMSO or enzalutamide (10 μM) for 10 days. The cells were then stained with Coomassie blue and photographed.

For cell proliferation assay, 1 × 10^4^ stable RNF8 knockdown or control LNCaP, LNCaP-EnzR, and 22Rv1 cells, 5 × 10^3^ stable RNF8 knockdown or control DU145 cells were divided into 96-well per well cultured in RPMI-1640 with 5% CSS and ethanol or DHT (10^−8^ M) or maintained with RPMI-1640 with 10% FBS with DMSO or enzalutamide (10 μM) with triple-well. The cell viability was detected by the CCK8 assay.

### In vivo tumor growth in xenograft models

The 5 × 10^6^ stable RNF8 knockdown and control 22Rv1 cells coated with 50% matrigel were injected subcutaneously in 4–5-week age male NOD/SCID mice (Charles River, Beijing). The tumor growth was monitored for 4 weeks as described previously [66]. The tumor volume was measured by the formula 0.5236 × *r*_1_^2^ × *r*_2_ (where *r*_1_ < *r*_2_) [16].

### Statistics

The number of replicates was presented by individual data points in each bar graph. Data were described as mean ± SEM and *p* values were determined by *t*-test, Mann–Whitney test, or one-way ANOVA test in Prism 9. RNF8 staining in clinical prostate, BPH, and PC samples was presented as median. RNF8, FASN, and ALDH1A3 staining in xenograft tumor sections was presented as mean. RNF8 staining characteristics of PC patients were assessed by the Chi-square test. The effect of clinicopathological characteristics and *RNF8* expression on the OS of PC patients was analyzed by ROC curve, UV COX, and MV COX regression in SPSS 26. The trend of RNF8 mRNA and protein expression with GS in PC was evaluated by the Jonckheere–Terpstra test in SPSS 26. The xenograft tumor volume and weight were presented as median. The significance is denoted as *P < 0.05, **P < 0.01, ***P < 0.001, ****P < 0.0001.

## Supplementary information

Supplementary Information

Original Data File

aj-checklist--CDDIS-21-3150RRR

## Data Availability

All data generated or analyzed during this study are included in this published article and its supplementary information files. The mRNA sequencing data generated during the current study is available in the NCBI GEO DataSets repository, https://www.ncbi.nlm.nih.gov/geo/query/acc.cgi?acc=GSE149581. The other datasets used and analyzed during the current study are available from the corresponding author on reasonable request.

## References

[1] Bray F, Ferlay J, Soerjomataram I, Siegel RL, Torre LA, Jemal A (2018). Global cancer statistics 2018: GLOBOCAN estimates of incidence and mortality worldwide for 36 cancers in 185 countries. CA Cancer J Clin.

[2] Scher HI, Sawyers CL (2005). Biology of progressive, castration-resistant prostate cancer: directed therapies targeting the androgen-receptor signaling axis. J Clin Oncol.

[3] Scher HI, Fizazi K, Saad F, Taplin ME, Sternberg CN, Miller K (2012). Increased survival with enzalutamide in prostate cancer after chemotherapy. N. Engl J Med.

[4] Teo MY, Rathkopf DE, Kantoff P (2019). Treatment of advanced prostate cancer. Annu Rev Med.

[5] Claessens F, Helsen C, Prekovic S, Van den Broeck T, Spans L, Van Poppel H (2014). Emerging mechanisms of enzalutamide resistance in prostate cancer. Nat Rev Urol.

[6] Wang Y, Chen J, Wu Z, Ding W, Gao S, Gao Y (2021). Mechanisms of enzalutamide resistance in castration-resistant prostate cancer and therapeutic strategies to overcome it. Br J Pharm.

[7] Linja MJ, Savinainen KJ, Saramaki OR, Tammela TL, Vessella RL, Visakorpi T (2001). Amplification and overexpression of androgen receptor gene in hormone-refractory prostate cancer. Cancer Res.

[8] Seruga B, Ocana A, Tannock IF (2011). Drug resistance in metastatic castration-resistant prostate cancer. Nat Rev Clin Oncol.

[9] Zhang L, Johnson M, Le KH, Sato M, Ilagan R, Iyer M (2003). Interrogating androgen receptor function in recurrent prostate cancer. Cancer Res.

[10] Prekovic S, van den Broeck T, Linder S, van Royen ME, Houtsmuller AB, Handle F (2018). Molecular underpinnings of enzalutamide resistance. Endocr Relat Cancer.

[11] Qu Y, Dai B, Ye D, Kong Y, Chang K, Jia Z (2015). Constitutively active AR-V7 plays an essential role in the development and progression of castration-resistant prostate cancer. Sci Rep..

[12] Wang J, Zou JX, Xue X, Cai D, Zhang Y, Duan Z (2016). ROR-gamma drives androgen receptor expression and represents a therapeutic target in castration-resistant prostate cancer. Nat Med.

[13] Bai S, Cao S, Jin L, Kobelski M, Schouest B, Wang X (2019). A positive role of c-Myc in regulating androgen receptor and its splice variants in prostate cancer. Oncogene..

[14] Bainbridge A, Walker S, Smith J, Patterson K, Dutt A, Ng YM (2020). IKBKE activity enhances AR levels in advanced prostate cancer via modulation of the Hippo pathway. Nucleic Acids Res.

[15] Metzger E, Wissmann M, Yin N, Muller JM, Schneider R, Peters AH (2005). LSD1 demethylates repressive histone marks to promote androgen-receptor-dependent transcription. Nature..

[16] Xu K, Shimelis H, Linn DE, Jiang R, Yang X, Sun F (2009). Regulation of androgen receptor transcriptional activity and specificity by RNF6-induced ubiquitination. Cancer Cell.

[17] Cano LQ, Lavery DN, Sin S, Spanjaard E, Brooke GN, Tilman JD (2015). The co-chaperone p23 promotes prostate cancer motility and metastasis. Mol Oncol.

[18] Sun S, Zhong X, Wang C, Sun H, Wang S, Zhou T (2016). BAP18 coactivates androgen receptor action and promotes prostate cancer progression. Nucleic Acids Res.

[19] Chene P (2003). Inhibiting the p53-MDM2 interaction: an important target for cancer therapy. Nat Rev Cancer.

[20] Xiao C, Wu G, Zhou Z, Zhang X, Wang Y, Song G (2019). RBBP6, a RING finger-domain E3 ubiquitin ligase, induces epithelial-mesenchymal transition and promotes metastasis of colorectal cancer. Cell Death Dis.

[21] Bhatnagar S, Gazin C, Chamberlain L, Ou J, Zhu X, Tushir JS (2014). TRIM37 is a new histone H2A ubiquitin ligase and breast cancer oncoprotein. Nature..

[22] Liu L, Wong CC, Gong B, Yu J (2018). Functional significance and therapeutic implication of ring-type E3 ligases in colorectal cancer. Oncogene..

[23] Huen MS, Grant R, Manke I, Minn K, Yu X, Yaffe MB (2007). RNF8 transduces the DNA-damage signal via histone ubiquitylation and checkpoint protein assembly. Cell..

[24] Mailand N, Bekker-Jensen S, Faustrup H, Melander F, Bartek J, Lukas C (2007). RNF8 ubiquitylates histones at DNA double-strand breaks and promotes assembly of repair proteins. Cell..

[25] Jacobs JJ (2012). Fusing telomeres with RNF8. Nucleus..

[26] Rai R, Li JM, Zheng H, Lok GT, Deng Y, Huen MS (2011). The E3 ubiquitin ligase Rnf8 stabilizes Tpp1 to promote telomere end protection. Nat Struct Mol Biol.

[27] David R (2011). Telomeres: fusing with RNF8. Nat Rev Mol Cell Biol.

[28] Plans V, Guerra-Rebollo M, Thomson TM (2008). Regulation of mitotic exit by the RNF8 ubiquitin ligase. Oncogene..

[29] Tripathi E, Smith S (2017). Cell cycle-regulated ubiquitination of tankyrase 1 by RNF8 and ABRO1/BRCC36 controls the timing of sister telomere resolution. EMBO J.

[30] Li L, Halaby MJ, Hakem A, Cardoso R, El Ghamrasni S, Harding S (2010). Rnf8 deficiency impairs class switch recombination, spermatogenesis, and genomic integrity and predisposes for cancer. J Exp Med.

[31] Li L, Guturi KKN, Gautreau B, Patel PS, Saad A, Morii M (2018). Ubiquitin ligase RNF8 suppresses Notch signaling to regulate mammary development and tumorigenesis. J Clin Invest.

[32] Lu LY, Wu J, Ye L, Gavrilina GB, Saunders TL, Yu X (2010). RNF8-dependent histone modifications regulate nucleosome removal during spermatogenesis. Dev Cell.

[33] Takano Y, Adachi S, Okuno M, Muto Y, Yoshioka T, Matsushima-Nishiwaki R (2004). The RING finger protein, RNF8, interacts with retinoid X receptor alpha and enhances its transcription-stimulating activity. J Biol Chem.

[34] Lee HJ, Li CF, Ruan D, Powers S, Thompson PA, Frohman MA (2016). The DNA damage transducer RNF8 facilitates cancer chemoresistance and progression through twist activation. Mol Cell.

[35] Wang S, Luo H, Wang C, Sun H, Sun G, Sun N (2017). RNF8 identified as a co-activator of estrogen receptor alpha promotes cell growth in breast cancer. Biochim Biophys Acta Mol Basis Dis.

[36] Halaby MJ, Hakem A, Li L, El Ghamrasni S, Venkatesan S, Hande PM (2013). Synergistic interaction of Rnf8 and p53 in the protection against genomic instability and tumorigenesis. PLoS Genet.

[37] Kuang J, Li L, Guo L, Su Y, Wang Y, Xu Y (2016). RNF8 promotes epithelial-mesenchymal transition of breast cancer cells. J Exp Clin Cancer Res.

[38] Zhou T, Yi F, Wang Z, Guo Q, Liu J, Bai N (2019). The functions of DNA damage factor RNF8 in the pathogenesis and progression of cancer. Int J Biol Sci.

[39] Ren L, Zhou T, Wang Y, Wu Y, Xu H, Liu J (2020). RNF8 induces beta-catenin-mediated c-Myc expression and promotes colon cancer proliferation. Int J Biol Sci.

[40] Uhlen M, Bjorling E, Agaton C, Szigyarto CA, Amini B, Andersen E (2005). A human protein atlas for normal and cancer tissues based on antibody proteomics. Mol Cell Proteom.

[41] Tang Z, Li C, Kang B, Gao G, Li C, Zhang Z (2017). GEPIA: a web server for cancer and normal gene expression profiling and interactive analyses. Nucleic Acids Res.

[42] Dardenne E, Beltran H, Benelli M, Gayvert K, Berger A, Puca L (2016). N-Myc induces an EZH2-mediated transcriptional program driving neuroendocrine prostate cancer. Cancer Cell.

[43] Roudier MP, Winters BR, Coleman I, Lam HM, Zhang X, Coleman R (2016). Characterizing the molecular features of ERG-positive tumors in primary and castration resistant prostate cancer. Prostate..

[44] Shiota M, Yokomizo A, Naito S (2011). Increased androgen receptor transcription: a cause of castration-resistant prostate cancer and a possible therapeutic target. J Mol Endocrinol.

[45] Zhang QC, Petrey D, Deng L, Qiang L, Shi Y, Thu CA (2012). Structure-based prediction of protein-protein interactions on a genome-wide scale. Nature..

[46] Zhang QC, Petrey D, Garzon JI, Deng L, Honig B (2013). PrePPI: a structure-informed database of protein-protein interactions. Nucleic Acids Res.

[47] Grad JM, Dai JL, Wu S, Burnstein KL (1999). Multiple androgen response elements and a Myc consensus site in the androgen receptor (AR) coding region are involved in androgen-mediated up-regulation of AR messenger RNA. Mol Endocrinol.

[48] Lee JG, Zheng R, McCafferty-Cepero JM, Burnstein KL, Nanus DM, Shen R (2009). Endothelin-1 enhances the expression of the androgen receptor via activation of the c-myc pathway in prostate cancer cells. Mol Carcinog.

[49] Dou Y, Milne TA, Tackett AJ, Smith ER, Fukuda A, Wysocka J (2005). Physical association and coordinate function of the H3 K4 methyltransferase MLL1 and the H4 K16 acetyltransferase MOF. Cell.

[50] Kouzarides T (2007). Chromatin modifications and their function. Cell..

[51] Pradeepa MM, Grimes GR, Kumar Y, Olley G, Taylor GC, Schneider R (2016). Histone H3 globular domain acetylation identifies a new class of enhancers. Nat Genet.

[52] Di Cerbo V, Mohn F, Ryan DP, Montellier E, Kacem S, Tropberger P (2014). Acetylation of histone H3 at lysine 64 regulates nucleosome dynamics and facilitates transcription. Elife..

[53] Wilson EM (2007). Muscle-bound? A tissue-selective nonsteroidal androgen receptor modulator. Endocrinology..

[54] Paschalis A, Sharp A, Welti JC, Neeb A, Raj GV, Luo J (2018). Alternative splicing in prostate cancer. Nat Rev Clin Oncol.

[55] Liu F, Wang L, Perna F, Nimer SD (2016). Beyond transcription factors: how oncogenic signalling reshapes the epigenetic landscape. Nat Rev Cancer.

[56] Deshar R, Yoo W, Cho EB, Kim S, Yoon JB (2019). RNF8 mediates NONO degradation following UV-induced DNA damage to properly terminate ATR-CHK1 checkpoint signaling. Nucleic Acids Res.

[57] Bohgaki T, Bohgaki M, Hakem R (2010). DNA double-strand break signaling and human disorders. Genome Integr.

[58] Wu J, Huen MS, Lu LY, Ye L, Dou Y, Ljungman M (2009). Histone ubiquitination associates with BRCA1-dependent DNA damage response. Mol Cell Biol.

[59] Richly H, Rocha-Viegas L, Ribeiro JD, Demajo S, Gundem G, Lopez-Bigas N (2010). Transcriptional activation of polycomb-repressed genes by ZRF1. Nature..

[60] Mattiroli F, Penengo L (2021). Histone ubiquitination: an integrative signaling platform in genome stability. Trends Gene.

[61] Debelouchina GT, Gerecht K, Muir TW (2017). Ubiquitin utilizes an acidic surface patch to alter chromatin structure. Nat Chem Biol.

[62] Takeyama K, Ito S, Yamamoto A, Tanimoto H, Furutani T, Kanuka H (2002). Androgen-dependent neurodegeneration by polyglutamine-expanded human androgen receptor in Drosophila. Neuron..

[63] Zhao Y, Lang G, Ito S, Bonnet J, Metzger E, Sawatsubashi S (2008). A TFTC/STAGA module mediates histone H2A and H2B deubiquitination, coactivates nuclear receptors, and counteracts heterochromatin silencing. Mol Cell.

[64] Hu R, Dunn TA, Wei S, Isharwal S, Veltri RW, Humphreys E (2009). Ligand-independent androgen receptor variants derived from splicing of cryptic exons signify hormone-refractory prostate cancer. Cancer Res.

[65] Kirkegaard T, Edwards J, Tovey S, McGlynn LM, Krishna SN, Mukherjee R (2006). Observer variation in immunohistochemical analysis of protein expression, time for a change?. Histopathology..

[66] Craft N, Shostak Y, Carey M, Sawyers CL (1999). A mechanism for hormone-independent prostate cancer through modulation of androgen receptor signaling by the HER-2/neu tyrosine kinase. Nat Med.

