# Supplementary material for: RNF8 up-regulates AR/ARV7 action to contribute to advanced prostate cancer progression
Source: Cell Death Dis. 2022 Apr 15;13(4):352. doi: 10.1038/s41419-022-04787-9 (PMC9012884; doi:10.1038/s41419-022-04787-9)
Supplement: Supplementary file 1 — Supplementary Information [file 41419_2022_4787_MOESM1_ESM.pdf]

RNF8 Up-regulates AR/ARV7 Action to Contribute to Advanced Prostate Cancer Progression

Tingting Zhou[1, 2], Shengli Wang[1], Xiaoyu Song[2], Wensu Liu[2], Fang Dong[3], Yunlong Huo[3], Renlong Zou[1], Chunyu Wang[1], Siyi Zhang[2], Wei Liu[1], Ge Sun[1], Lin Lin[1], Kai Zeng[1], Xiang Dong[2], Qiqiang Guo[2], Fei Yi[2], Zhuo Wang[2], Xiaoman Li[2], Bo Jiang[2], Liu Cao[2*], Yue Zhao[1*]

[1] Department of Cell Biology, Key Laboratory of Cell Biology, Ministry of Public Health, and Key Laboratory of Medical Cell Biology, Ministry of Education, School of Life Sciences, China Medical University, Shenyang, Liaoning Province, 110122, PR China.
[2] College of Basic Medical Science, Institute of Health Sciences, Key Laboratory of Cell Biology of Ministry of Public Health, Key Laboratory of Medical Cell Biology of Ministry of Education, Liaoning Province Collaborative Innovation Center of Aging Related Disease Diagnosis and Treatment and Prevention, China Medical University, Shenyang, Liaoning Province, 110122, PR China.
[3] Department of Pathology, Shengjing Hospital of China Medical University, Shenyang, Liaoning Province, 110004, PR China

[*] To whom correspondence should be addressed.
Yue Zhao. Department of Cell Biology, Key laboratory of Cell Biology, Ministry of Public Health, and Key laboratory of Medical Cell Biology, Ministry of Education, School of Life Sciences, China Medical University, No.77 Puhe Road, Shenyang North New Area, Shenyang City 110122, Liaoning Province, China.
Tel: +86 24 31939077; Fax: +86 24 31939077; Email: yzhao30@cmu.edu.cn
ORCID: https://orcid.org/0000-0001-8983-0024
Liu Cao. College of Basic Medical Science, Institute of Health Sciences, Key Laboratory of Cell Biology of Ministry of Public Health, Key Laboratory of Medical Cell Biology of Ministry of Education, Liaoning Province Collaborative Innovation Center of Aging Related Disease Diagnosis and Treatment and Prevention, China Medical University, No.77 Puhe Road, Shenyang North New Area, Shenyang City 110122, Liaoning Province, China. Tel: +86 24 31939636; Email: lcao@cmu.edu.cn
ORCID: https://orcid.org/0000-0001-6471-1993

SUPPLEMENTARY FIGURE LEGENDS

Supplementary Fig. 1 The H&E staining and the negative control staining of PC tissues. A The representative images for the corresponding sections of Normal, GS=5, GS=7, and GS=9 in Figure 1C performed with H&E staining. **B** The representative images for the negative control (without primary antibody) of the GS=7 PC section. **C** The representative images for the xenograft tumor sections negatively stained with rabbit and mouse IgG. Scale bar, 50 μm.

Supplementary Fig. 2 The resistance efficacy of LNCaP-EnzR cells to enzalutamide. LNCaP and LNCaP-EnzR cells were treated with vehicle or enzalutamide (10 μM) for 0, 1, 2, 3, 4, 5, 6, 7 days and performed for the CCK8 assay. Data are means ± SEM (n=3). *, $P<0.05$; ns=no significant change (two-sided upaired *t*-test).

Supplementary Fig. 3. RNF8 enhances *AR* transcription dependent on its E3 ligase activity. A The efficacy of siRNA targeting RNF8 in LNCaP, 22Rv1, and LNCaP-EnR cells was determined by qRT-PCR (n=3). **B** The transcription levels of *AR* pre-mRNA were detected by three pairs of primers in control and RNF8 silencing LNCaP (n=3), 22Rv1 (n=3), and LNCaP-EnR (n=3) cells. **C** The transcription levels of *AR* and *ARV7* in control and RNF8 silenced LNCaP (n=3), 22Rv1 (n=3), and LNCaP-EnR (n=3) cells. **D** The transcription levels of *AR* and *ARV7* in 22Rv1 cells with RNF8 overexpression (n=3). **E** The mRNA expressions of AR and ARV7 were detected in 22Rv1 cells transfected with RNF8 or RNF8C403S (n=3). Data of the qRT-PCR are presented as the ratio normalized to *PPIA* (means ± SEM). The

statistical analysis is One-way ANOVA for (A) and (B); two-sided upaired *t*-test for (C), (D), and (E). *, P<0.05; **, P<0.01; ***, P<0.001; ****, P<0.0001.

Supplementary Fig. 4 RNF8 does not affect the protein stability of AR/ARV7. A, B LNCaP cells were transfected with siCtrl/siRNF8 or vector/Flag-RNF8 for 48 hours and conducted for western blot using indicated antibodies. **C** 22Rv1 cells were overexpressed with vector or Flag-RNF8. 24 hours later, cells were treated with CHX (10 μg/ml) for 0, 2, 4, 6 hours and harvested for western blot with indicated antibodies. **D** Statistic analysis of AR, AR-Vs, and ARV7 in Flag-RNF8 group versus vector group in (C). Data are presented as means ± SEM (n=3) from three independent experiments. ns=no significant change (two-sided upaired *t*-test). **E** 22Rv1 cells were silenced with control siRNA or siRNA targeting RNF8 for 48 hours. The cells were harvested after vehicle or MG132 (20 μM) treated for 30 minutes and were immunoblotted for AR, ARV7, and RNF8. Numbers under the western blot bands indicate the relative grey value normalized to the internal control (α-tubulin or β-actin).

Supplementary Fig. 5 RNF8 does not influence the mRNA and protein levels of c-Myc. A-C LNCaP and 22Rv1 cells were transfected with control siRNA or siRNA targeting RNF8 for 48 hours. Then, the cells were harvested for western blot with RNF8 and c-Myc antibodies, or the qRT-PCR with specific *RNF8* and *MYC* primers. Data of the qRT-PCR are presented as the ratio normalized to *RPS18* or *ACTB* (means ± SEM, n=6). ****, P<0.0001; ns=no significant change (two-sided upaired *t*-test). Numbers under the western blot bands indicate the relative grey value normalized to the internal control (β-actin or α-tubulin).

Supplementary Fig. 6 The transcription of AR target genes is influenced by RNF8 silencing. A-C 22Rv1 cells were transfected with siCtrl or siRNF8. After 24 hours, the cells were treated for vehicle or DHT (10^{-8} M) for 24 hours. The cells were harvested for the qRT-PCR with indicated primers. **D-E** The mRNA level of *KLK3* in 22Rv1 cells transfected with siCtrl/siRNF8 or RNF8/RNF8-C403S and treated with or without DHT (10^{-8} M) or enzalutamide (10 μM) for 24 hours. Data are presented as the ratio normalized to *RPS18* or *ACTB* (means ± SEM, n=3). *, P<0.05; **, P<0.01; ***, P<0.001; ****, P<0.0001 (two-sided upaired *t*-test).

Supplementary Fig. 7 The efficacy of RNF8 silencing in LNCaP and 22Rv1 cells and xenograft tumors. A, B LNCaP and 22Rv1 cells were infected with lentivirus carrying control or RNF8 targeting shRNA and screened by puromycin for one week. The cell lysates were conducted for western blot with RNF8 antibody. **C** The RNF8 expression levels were detected in lysates of control and RNF8 silencing xenograft tumor tissues by western blot with indicated antibodies. Numbers below the western blot result indicate the relative grey value normalized to the internal control (β-actin or GAPDH).

Supplementary Figure 1

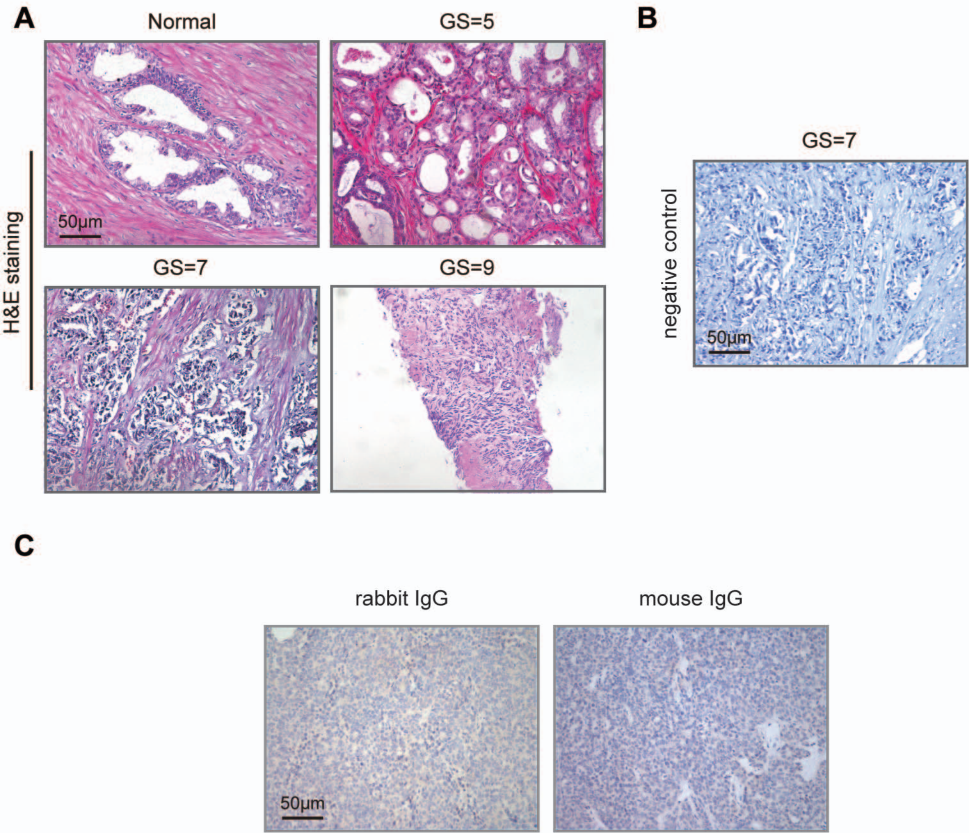

Supplemenatry Figure 2

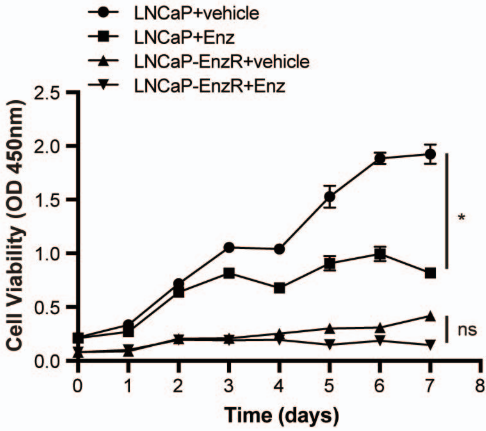

Supplementary Figure 3

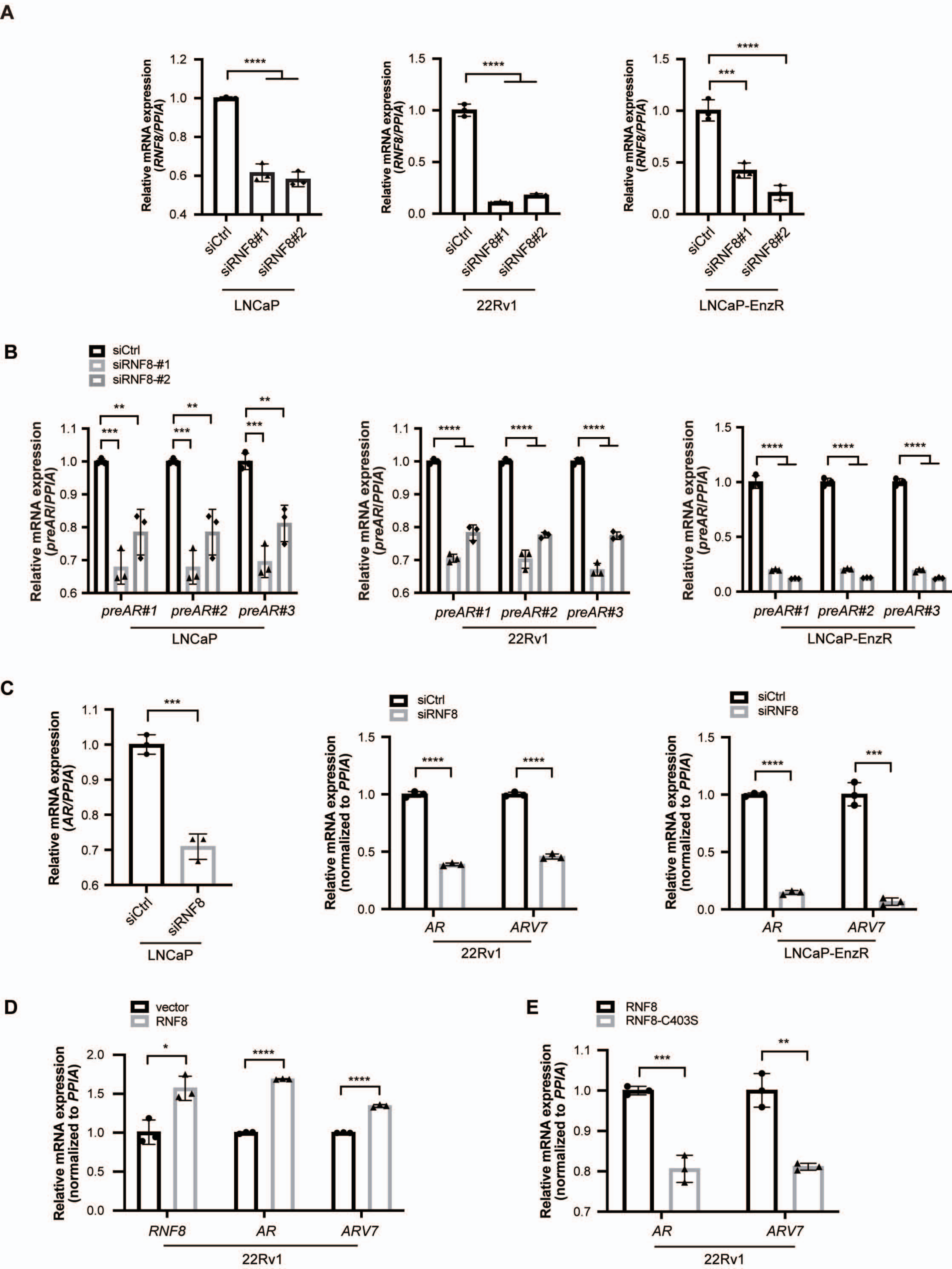

Supplementary Figure 4

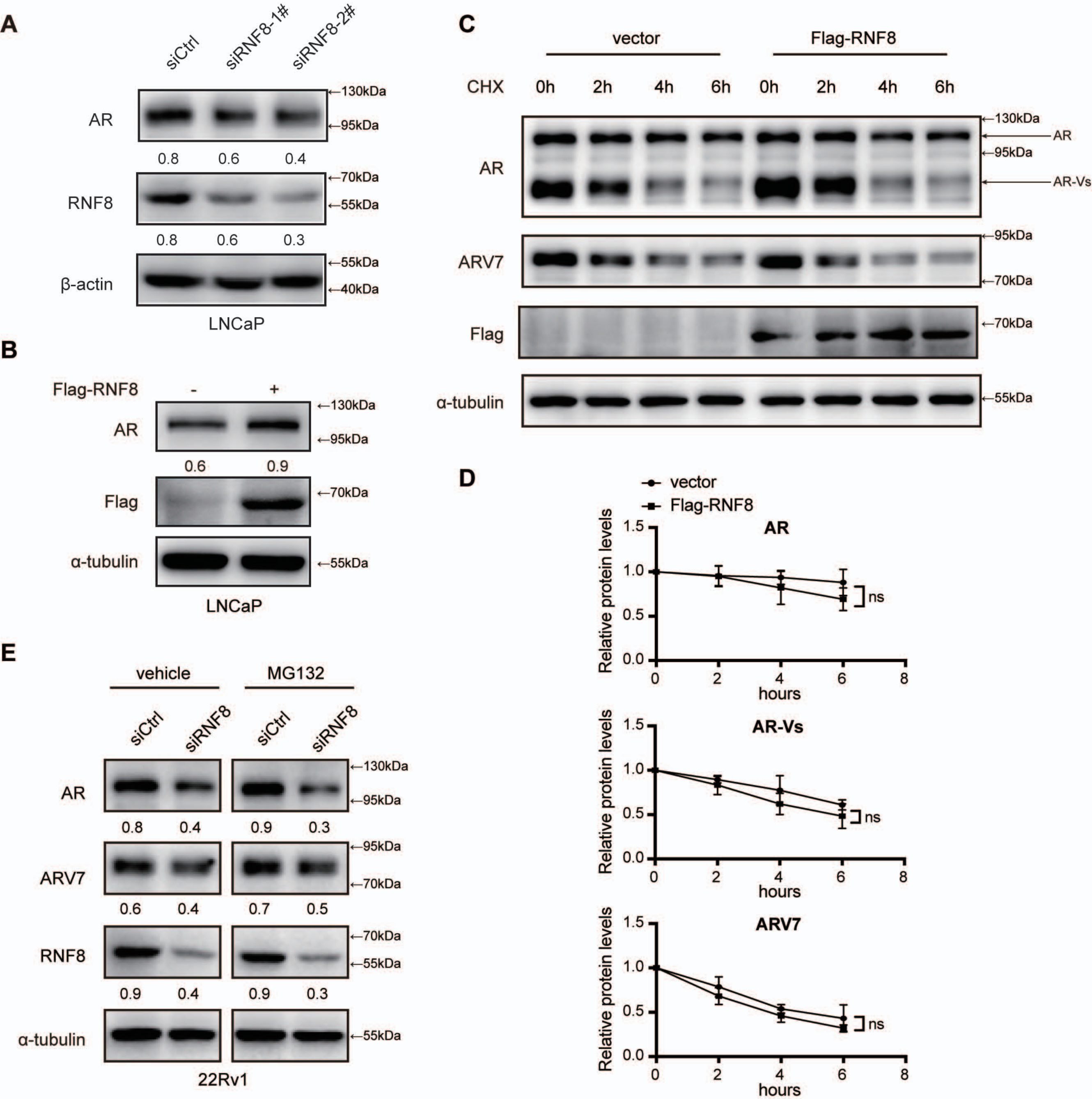

Supplementary Figure 5

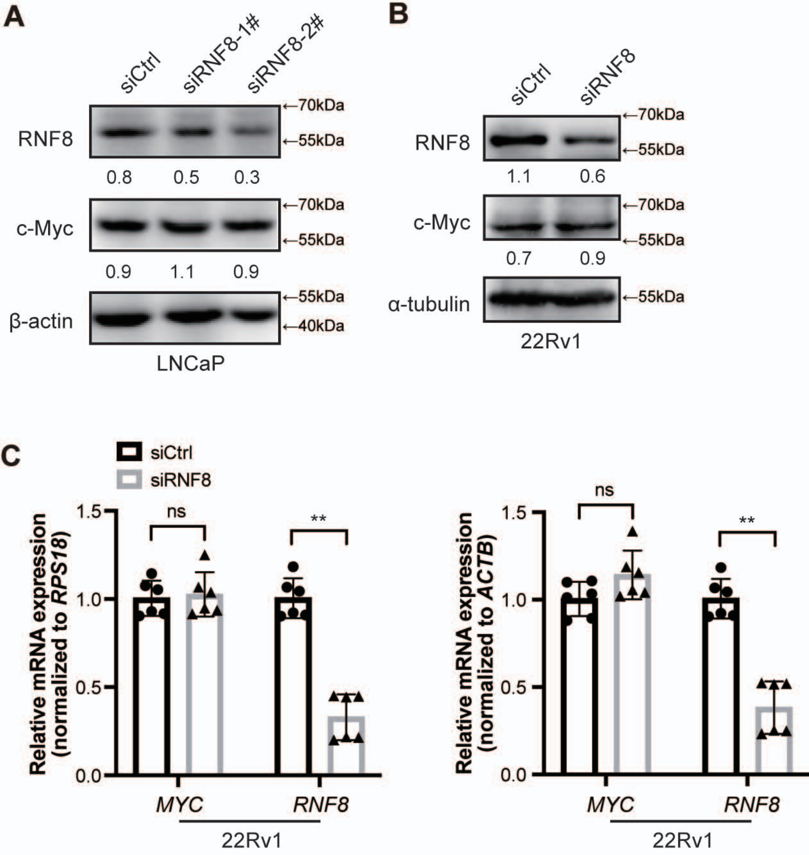

Supplementary Figure 6

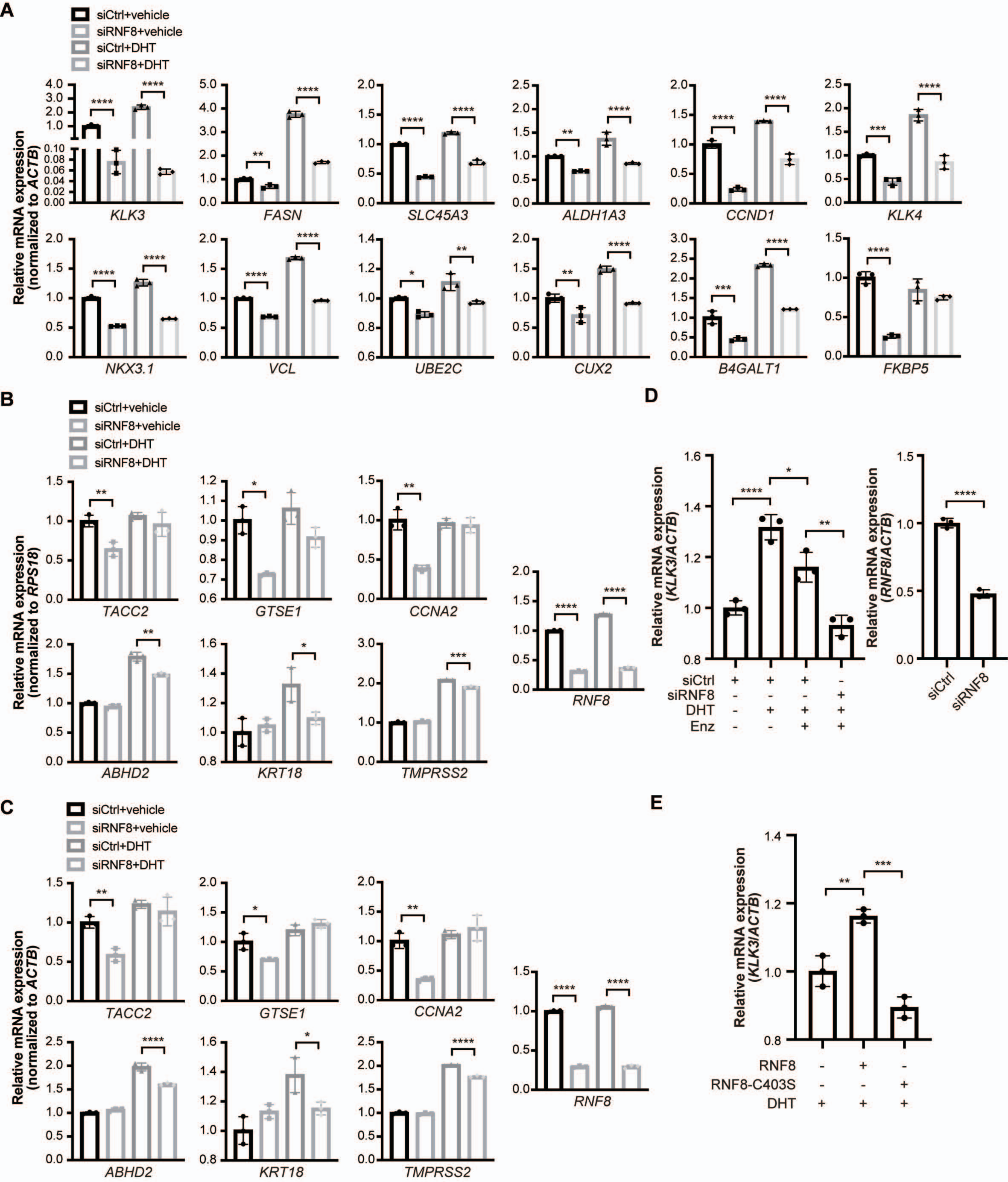

Supplementary Figure 7

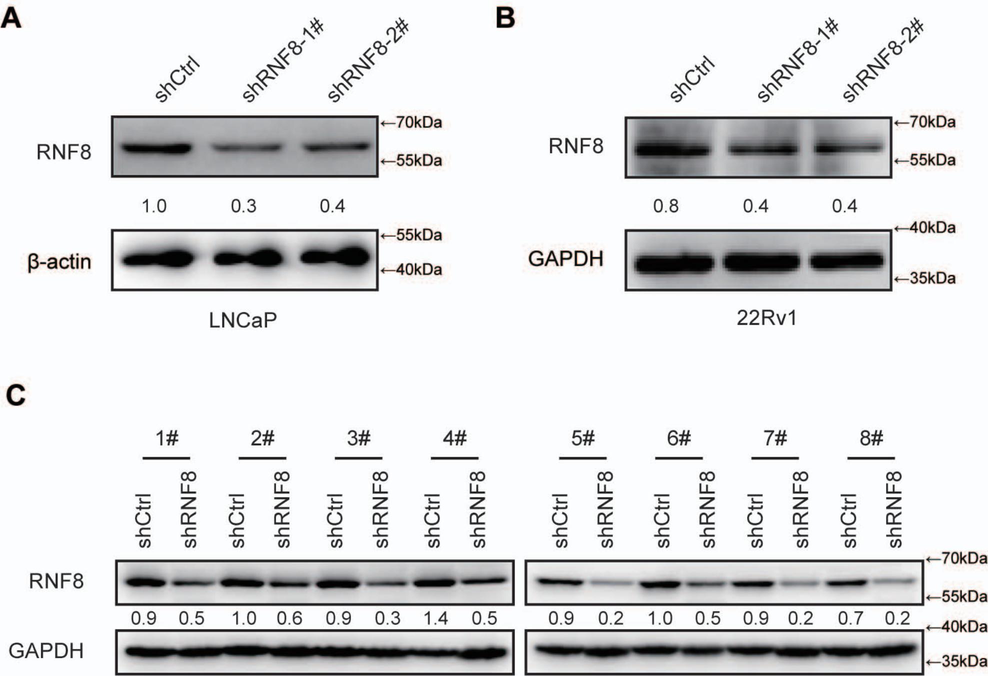

Supplementary Table 1. The evaluation for the effect of *RNF8* mRNA expression and clinicopathological variables on the overall survival (OS) of prostate cancer patients

A. The ROC analysis for the effect of *RNF8* mRNA expression, age, Gleason score, and PSA values on the OS of prostate cancer patients

Area Under the Curve

				Asymptotic 95% Confidence Interval	
	**Area**	**Std. Error**[a]	**Asymptotic Sig.**[b]	**Lower Bound**	**Upper Bound**
RNF8	0.687	0.074	**0.034**	0.542	0.832
**age**	0.540	0.099	0.648	0.347	0.734
**Gleason score**	0.679	0.078	**0.043**	0.527	0.831
**PSA**	0.591	0.092	0.378	0.410	0.772

a. Under the nonparametric assumption
b. Null hypothesis: true area = 0.5

B. The univariate COX (UV COX) regression analysis for the effect of *RNF8* mRNA expression, Gleason score, TNM, race, additional pharmaceutical therapy, and additional radiation therapy on the OS of prostate cancer patients

Variables in the Equation

	B	SE	Wald	df	Sig.	Exp(B)	95.0% CI for Exp(B)	
							Lower	Upper
RNF8	-0.201	0.096	4.388	1	**0.036**	0.818	0.678	0.987
**Gleason score**	0.031	0.097	0.104	1	0.747	1.032	0.854	1.247
**TNM**			2.671	4	0.614			
TNM(1)	-0.588	1.225	0.230	1	0.631	0.556	0.050	6.127
TNM(2)	1.386	1.225	1.281	1	0.258	4.000	0.363	44.113
TNM(3)	-11.808	876.011	0.000	1	0.989	0.000	0.000	
TNM(4)	-0.304	0.837	0.132	1	0.717	0.738	0.143	3.804
**race**			15.217	4	**0.004**			
race(1)	-0.273	1.039	0.069	1	0.793	0.761	0.099	5.834
race(2)	-0.204	1.002	0.042	1	0.838	0.815	0.114	5.814
race(3)	-0.283	1.042	0.074	1	0.786	0.754	0.098	5.807
race(4)	-0.819	1.013	0.654	1	0.419	0.441	0.061	3.210
**additional pharmaceutical therapy**	0.750	0.286	6.865	1	**0.009**	2.116	1.208	3.708
**additional radiation therapy**	-0.219	0.273	0.641	1	0.423	0.804	0.470	1.373

C. The multivariate COX (MV COX) regression analysis for the effect of *RNF8* mRNA expression, race, and additional pharmaceutical therapy on the OS of prostate cancer patients

Variables in the Equation

	**B**	**SE**	**Wald**	**df**	**Sig.**	**Exp(B)**	**95.0% CI for Exp(B)**	
							**Lower**	**Upper**
RNF8	-0.663	0.294	5.092	1	**0.024**	0.515	0.290	0.917
**race**			5.027	3	0.170			
race(1)	-1.486	1.058	1.972	1	0.160	0.226	0.028	1.800
race(2)	-2.709	1.489	3.310	1	0.069	0.067	0.004	1.233
race(3)	-2.083	1.143	3.320	1	0.068	0.124	0.013	1.171
**additional_pharmaceutical_therapy**	1.145	0.321	12.737	1	**0.000**	3.143	1.676	5.895

Supplementary Table 2. The trend of *RNF8* mRNA/RNF8 protein expression with Gleason score in prostate cancer

A. The hypothesis test for the distribution of *RNF8* mRNA/RNF8 protein expression and Gleason score

Hypothesis Test Summary

Null Hypothesis	Test	Sig.	Decision
The distribution of Coping with *RNF8* mRNA expression is the same across categories of Gleason score	Independent-Samples Jonckheere-Terpstra Test for Ordered Alternatives	**0.000**	Reject the null hypothesis

Asymptotic significances are displayed. The significant level is 0.05.

B. The summary of the Independent-Samples Jonckheere-Terpstra test for ordered alternatives

Jonckheere-Terpstra Test [a]

	***RNF8* mRNA expression**	**RNF8 protein expression**
**Number of Levels in Gleason score**	3	3
**N**	440	105
**Observed J-T Statistic**	36101.000	2489.500
**Mean J-T Statistic**	27987.500	1811.500
**Std. Deviation of J-T Statistic**	1387.022	169.038
**Std. J-T Statistic**	5.850	4.011
**Asymp. Sig. (2-tailed)**	**0.000**	**0.000**

a. Grouping Variable: Gleason score

Supplementary Table 3. RNF8 staining characteristics of prostate cancer patients

differentiation	**RNF8** (155)			
	Low (%)	High (%)	total	p
Normal+BPH	46 (92.0)	4 (8.0)	50	
GS<7	27 (81.8)	6 (18.2)	33	**0.000**
GS≥7	28 (38.9)	44 (61.1)	72	

BPH=benign prostatic hyperplasia
Chi-sequare test were used.

Supplementary Table 4. AR-induced genes differentially expressed and the genome-wide impact of RNF8 in the absence or presence of DHT in 22Rv1 cells

Name	Gene ID	shCtrl-DHT/shCtrl-Veh		shRNF8-DHT/shCtrl-DHT		shRNF8-Veh/shCtrl-Veh	
		Fold change	p-value	Fold change	p-value	Fold change	p-value
ACADL	33	2.81	0.00	2.37	0.02	9.97	0.00
CTF1	1489	3.92	0.00	-31.33	0.00	-8.00	0.00
CYP2E1	1571	3.82	0.02	-48.33	0.00	-12.67	0.00
ACSL3	2181	2.52	0.00	2.06	0.00	3.97	0.00
GLUL	2752	1.62	0.04	1.94	0.04	1.84	0.04
GOLGA4	2803	1.59	0.03	4.97	0.00	9.72	0.00
GTF2A2	2958	1.51	0.04	3.24	0.00	7.73	0.01
H2AFZ	3015	1.82	0.01	-3.16	0.01	-2.86	0.00
HPD	3242	3.07	0.02	-290.67	0.00	-94.67	0.00
ITGA4	3676	1.77	0.04	-7.67	0.00	-4.33	0.00
ITGA9	3680	3.90	0.00	-50.67	0.00	-13.00	0.00
LAMA3	3909	2.02	0.03	9.26	0.00	-13.67	0.00
NDUFC2	4718	2.43	0.00	3.28	0.04	5.26	0.01
PER1	5187	1.79	0.01	3.33	0.00	3.25	0.03
RGS2	5997	1.82	0.00	1.96	0.01	4.09	0.00
SOX11	6664	1.66	0.04	-10.59	0.02	-22.67	0.00
SPOCK1	6695	4.13	0.00	10.26	0.00	82.72	0.00
VEGFC	7424	2.08	0.02	-34.27	0.01	-116.67	0.00
ZBTB16	7704	1.78	0.04	3.28	0.01	6.26	0.00
PRSS12	8492	1.55	0.01	-82.00	0.00	-53.00	0.00
KMO	8564	1.97	0.05	-24.33	0.00	-12.33	0.00
DBF4	10926	1.50	0.00	3.55	0.00	5.20	0.03
TBC1D8	11138	2.88	0.00	2.27	0.01	4.03	0.01
ELL2	22936	1.64	0.02	2.48	0.01	2.45	0.00
ABCA5	23461	1.66	0.00	5.86	0.00	13.38	0.00
STEAP1	26872	5.21	0.00	6.11	0.00	21.60	0.01
B3GAT1	27087	5.94	0.01	-126.67	0.00	-21.33	0.00
IL20RA	53832	1.91	0.01	-169.67	0.00	-89.00	0.00
WDYHV1	55093	1.60	0.02	3.24	0.00	6.60	0.02
RIC8B	55188	1.66	0.01	3.27	0.02	9.62	0.00
JAM3	83700	1.72	0.00	-213.00	0.00	-124.00	0.00
MEGF10	84466	2.25	0.02	-21.00	0.00	-9.33	0.00

TUBA1C	84790	1.62	0.00	-2.21	0.03	-1.68	0.00
TBRG1	84897	3.72	0.00	2.14	0.00	6.48	0.00
ACPT	93650	2.84	0.01	-80.33	0.00	-28.33	0.00
TUBA3D	113457	3.53	0.03	-82.33	0.00	-23.33	0.00
TMEM132B	114795	2.30	0.01	-7.67	0.00	-3.33	0.00
GBP5	115362	2.79	0.00	-298.00	0.00	-106.67	0.00
MBOAT2	129642	5.96	0.00	2.42	0.01	13.07	0.04
C2orf76	130355	3.93	0.03	-712.33	0.00	-181.33	0.00
EGFLAM	133584	2.05	0.05	-13.67	0.00	-6.67	0.00
SGOL1	151648	1.76	0.01	9.32	0.00	32.41	0.00
SGK223	157285	2.14	0.00	2.34	0.03	3.17	0.02
PHOSPHO1	162466	2.97	0.03	-173.33	0.00	-58.33	0.00
TUBB	203068	1.52	0.02	-2.23	0.00	-2.45	0.00
STEAP2	261729	2.18	0.02	4.17	0.00	15.07	0.04
ENO4	387712	1.90	0.02	-44.33	0.00	-23.33	0.00
NDUFC2-KCTD14	100532726	3.08	0.01	-110.72	0.00	-35.92	0.00
BMP2	650	-2.17	0.03	12.93	0.00	8.35	0.04
CACNB2	783	-1.52	0.02	11.08	0.00	6.09	0.02
CHRNA7	1139	-1.53	0.05	-18.74	0.01	-127.67	0.00
HOXD13	3239	-3.05	0.02	-12.67	0.00	-38.67	0.00
INHBA	3624	-6.39	0.00	15.23	0.00	12.63	0.00
IRS1	3667	-2.17	0.01	-3.12	0.01	-4.61	0.03
KCNA7	3743	-1.68	0.02	-69.67	0.00	-117.33	0.00
KCNH1	3756	-7.00	0.01	-2.00	0.00	-14.00	0.00
MST1	4485	-1.67	0.01	4.52	0.02	-239.08	0.00
NTSR1	4923	-2.02	0.04	-15.67	0.00	-31.67	0.00
PRRX1	5396	-1.78	0.02	-12.51	0.01	-81.33	0.00
RDH5	5959	-2.00	0.01	38.41	0.00	42.51	0.00
SH3GL3	6457	-2.86	0.00	-38.33	0.00	-109.67	0.00
ST3GAL1	6482	-2.06	0.02	5.98	0.00	3.63	0.01
SLC7A5	8140	-1.66	0.05	-2.44	0.01	-4.23	0.00
IRS2	8660	-1.77	0.00	-1.54	0.02	-2.45	0.00
NMI	9111	-1.72	0.04	-77.33	0.00	-133.33	0.00
CD83	9308	-1.70	0.01	-47.00	0.00	-79.67	0.00
CREB5	9586	-1.55	0.02	20.83	0.00	10.35	0.00
PCDHGA8	9708	-2.74	0.04	-7.67	0.00	-21.00	0.00
TNFSF15	9966	-2.44	0.02	5.60	0.01	5.56	0.01

EMILIN1	11117	-1.71	0.02	-58.33	0.00	-100.00	0.00
MRAS	22808	-1.69	0.00	-9.06	0.04	-20.00	0.02
NFASC	23114	-1.54	0.02	5.61	0.00	5.26	0.00
LRRC6	23639	-1.88	0.05	-54.67	0.00	-103.00	0.00
NPAP1	23742	-1.63	0.05	-10.00	0.00	-16.33	0.00
AIPL1	23746	-1.58	0.03	-32.00	0.00	-50.67	0.00
TOX3	27324	-2.47	0.00	5.16	0.01	3.59	0.00
PSAT1	29968	-1.60	0.03	-3.70	0.00	-4.99	0.01
TLR7	51284	-1.86	0.01	-31.67	0.00	-59.00	0.00
ATP8A2	51761	-2.67	0.02	-6.00	0.00	-16.00	0.00
EPB41L4A-AS2	54508	-2.91	0.02	-35.33	0.00	-102.67	0.00
SYTL2	54843	-1.67	0.03	34.45	0.00	28.44	0.00
KLHDC8A	55220	-3.77	0.02	-14.67	0.00	-55.33	0.00
PCDHA11	56138	-1.92	0.01	-397.00	0.00	-762.67	0.00
VN1R1	57191	-3.12	0.04	-13.67	0.00	-42.67	0.00
ZNF624	57547	-1.89	0.02	-32.00	0.00	-60.33	0.00
BEGAIN	57596	-1.68	0.05	-61.67	0.00	-103.33	0.00
KIAA1462	57608	-3.12	0.02	-5.67	0.00	-17.67	0.00
BRINP2	57795	-3.59	0.04	-10.67	0.01	-38.33	0.00
OXCT2	64064	-2.01	0.05	-17.88	0.01	-114.33	0.00
ZSCAN31	64288	-1.84	0.03	3.92	0.02	-119.00	0.00
IKZF4	64375	-1.66	0.01	9.39	0.00	4.83	0.00
DUSP26	78986	-3.14	0.01	-48.33	0.00	-152.00	0.00
NEIL1	79661	-1.80	0.01	8.59	0.00	4.16	0.00
EFCC1	79825	-3.42	0.03	-24.33	0.00	-83.33	0.00
APOL6	80830	-5.08	0.02	-3.58	0.02	-26.88	0.00
DISP1	84976	-2.70	0.00	9.56	0.01	5.52	0.02
SMIM3	85027	-1.91	0.00	-117.67	0.00	-224.67	0.00
ZNF837	116412	-1.52	0.01	-195.00	0.00	-77.85	0.00
HIST2H3C	126961	-1.60	0.00	-16.77	0.01	-108.67	0.00
TMEM182	130827	-1.59	0.01	5.39	0.05	-208.67	0.00
PRIMA1	145270	-2.37	0.02	-48.33	0.00	-114.67	0.00
CREB3L4	148327	-1.75	0.01	3.25	0.00	1.83	0.02
IL23R	149233	-2.10	0.01	-10.33	0.00	-21.67	0.00
ADAMTS19	171019	-1.59	0.00	-28.33	0.00	-45.00	0.00
UNC13D	201294	-2.88	0.03	191.86	0.00	-16.33	0.00
CRB2	286204	-2.95	0.00	-6.33	0.00	-18.67	0.00

HIST2H3A	333932	-1.60	0.00	-16.77	0.01	-108.67	0.00
MS4A10	341116	-1.70	0.01	-48.00	0.00	-81.67	0.00
FBLL1	345630	-2.37	0.00	-412.00	0.00	-977.00	0.00
TNFAIP8L3	388121	-2.44	0.00	-6.00	0.00	-14.67	0.00
C15orf59	388135	-1.91	0.03	-131.33	0.00	-250.67	0.00
GLTPD2	388323	-1.84	0.02	-290.67	0.00	-534.00	0.00
TMEM238	388564	-1.61	0.03	-45.23	0.02	-33.65	0.03
HRNR	388697	-2.07	0.01	-9.33	0.00	-19.33	0.00
LINC00999	399744	-1.89	0.02	-11.28	0.03	-138.67	0.00
KRTAP5-2	440021	-2.57	0.05	-147.00	0.00	-378.33	0.00
NKX2-4	644524	-2.71	0.01	-42.67	0.00	-115.67	0.00
PABPC1L2B	645974	-2.68	0.01	-79.67	0.00	-213.33	0.00
GOLGA6L6	727832	-1.81	0.03	-17.67	0.00	-32.00	0.00
SLC25A21-AS1	100129794	-2.45	0.02	-241.61	0.00	-594.67	0.00

Supplementary Table 5. The mRNA expression correlation between *RNF8* and its regulated genes in prostate cancer samples of TCGA

Gene name	NCBI Gene ID	Correlation
KLK3	354	negative
FASN	2194	no
SLC45A3	85414	no
ALDH1A3	220	positive
CCND1	595	positive
KLK4	9622	negative
NKX3.1	4824	positive
VCL	7414	positive
CCNA2	890	positive
FKBP5	2289	positive
B4GALT1	2683	positive
TACC2	10579	positive
UBE2C	11065	positive
GTSE1	51512	positive
CUX2	23316	positive
ABHD2	11057	positive
KRT18	3875	no
TMPRSS2	7113	positive

Supplementary Table 6. qRT-PCR primers for genes as indicated

Name	Sense (5'-3')	Anti-sense (5'-3')
RNF8	CCGCTGCTCTGAAGGTCAACTG	GTGCCGTGAATCCTGGTGGTG
preAR#1	GGTGAGCAGAGTGCCCTATC	GCCGCTAGATACCCCAGAAC
preAR#2	TTACGGGGACATGCGGTAAG	AGGGGCAATCTGAGTGTTCG
preAR#3	CCCAGTCCCACTTGTGTCAA	TAGGAGCCGCTAGATACCCC
AR	GCTGCTCCGCTGACCTTAA	CGACACTGCCTTACACAACTC
ARV7	CTACTCCGGACCTTACGGGGACATGCG	TGCCAACCCGGAATTTTTCTCCC
KLK3	CACCTGCTCGGGTGATTCTG	CCACTTCCGGTAATGCACCA
FASN	TTGTGGTCTTCTCCTCTGTGA	CGTTGGTGCTCATCGTCTC
UBE2C	TAAAGGAGCTGAGCCGAGCG	CATATACTGTTCCAGCTGCTCCAT
SLC45A3	GAGCCGAGACGAAGCAGTT	TTAGCAGGTTGACCAGCAAGA
ALDH1A3	TGACATTGACCGTGAGATTCG	CTATGCTGTTGTGGCGTTAGA
ABHD2	AGTTCGTGTCCTAATGGTCTCT	CGCAGATGTTCAGCAATGTTC
CUX2	GAAGCCAACCGTGAAGGAAC	CCTCAGCATCCTCCTCCATC
TMPRSS2	TGAAAGCGGGTGTGAGGAGC	TGGTGGTGACCCTGAGTTCAA
CCND1	GGATGCTGGAGGTCTGCGA	AGAGGCCACGAACATGCAAG
ACPP	ACTGGTCCACGGAGTGTATC	CAGGTGAAGAGGTAGGAATTGC
FKBP5	CGCAGGATATACGCCAACAT	CTTGCCCATTGCTTTATTGG
CCNA2	GAATGAGACCCTGCATTTGG	GCCCACAAGCTGAAGTTTTC
TACC2	CTGGAGAGGCAGGTGTCAGA	TTGCTCCGTGTTCAAGTGCT
B4GALT1	GGGAGGAGAAGATGATGACATT	TTGGGCGAGATATAGACATGC
NKX3.1	GCTCACGTCCTTCCTCATCCA	AGTGCCTTTCTGGCTCGGTC
RPS18	GTAACCCGTTGAACCCCATT	CCATCCAATCGGTAGTAGCG

Supplementary Table 7. siRNA sequences against RNF8

Name	Sense (5'-3')	Antiense (5'-3')
siRNF8#1	GGACAAUUAUGGACAACAAdTdT	UUGUUGUCCAUAUUGUCCdTdT
siRNF8#2	GCUUCUUCGUCACAGGAGAdTdT	UCUCCUGUGACGAAGAAGCdTdT

Supplementary Table 8. shRNA target sites of RNF8

Name	Target	Start site
shRNF8#1	AAGAATTAGAGCAGACCAA	1257
shRNF8#2	GAGCAACTAGAGAAGACTT	1079

Supplementary Table 9. The interclass correlation coefficient of the histochemistry evaluation of RNF8 expression in prostate cancer tissues by two observers

	Cases	ICCC
**Overall**	156	0.906
**Observer 1**	156	0.901
**Observer 2**	156	0.874
