# Supplementary material for: RNF8 up-regulates AR/ARV7 action to contribute to advanced prostate cancer progression
Source: Cell Death Dis. 2022 Apr 15;13(4):352. doi: 10.1038/s41419-022-04787-9 (PMC9012884; doi:10.1038/s41419-022-04787-9)
Supplement: Supplementary file 2 — Original Data File [file 41419_2022_4787_MOESM2_ESM.pdf]

## Original data file 1

**A**

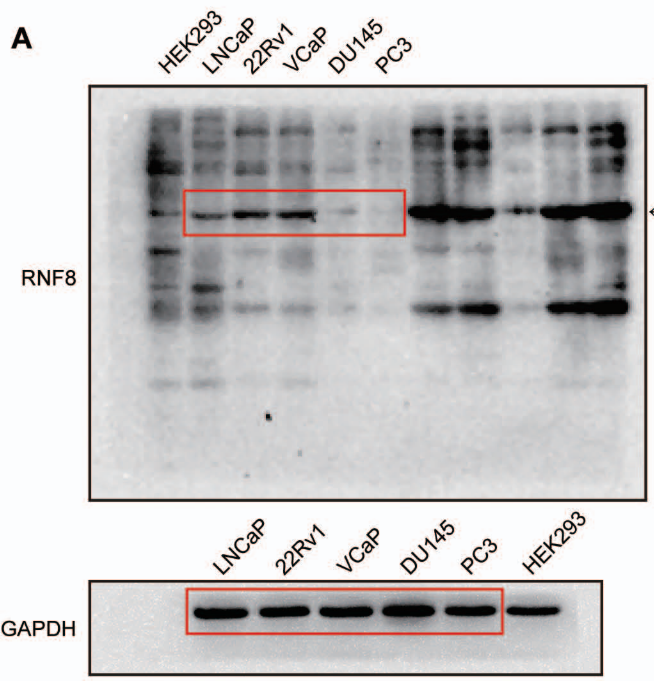


Figure 1E

**B**

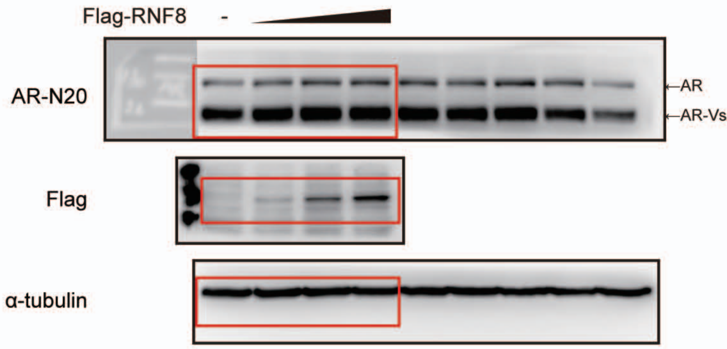


Figure 2F

**C**

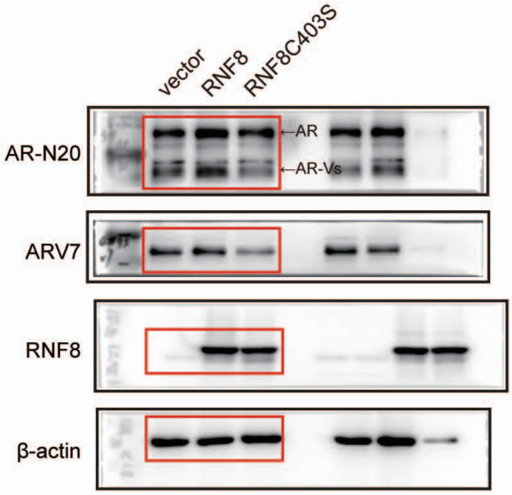


Figure 2H

**D**

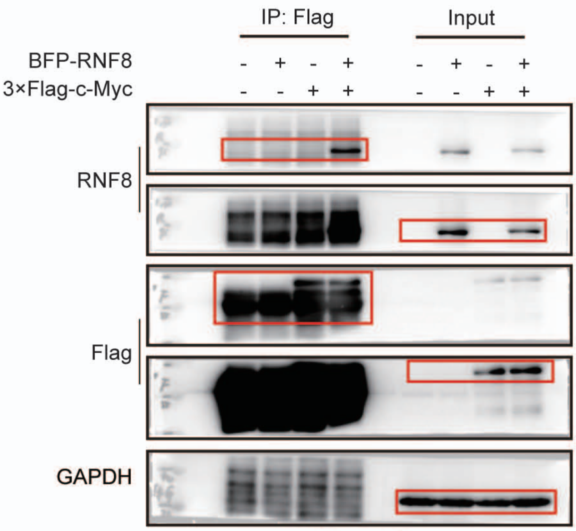


Figure 3B

**E**

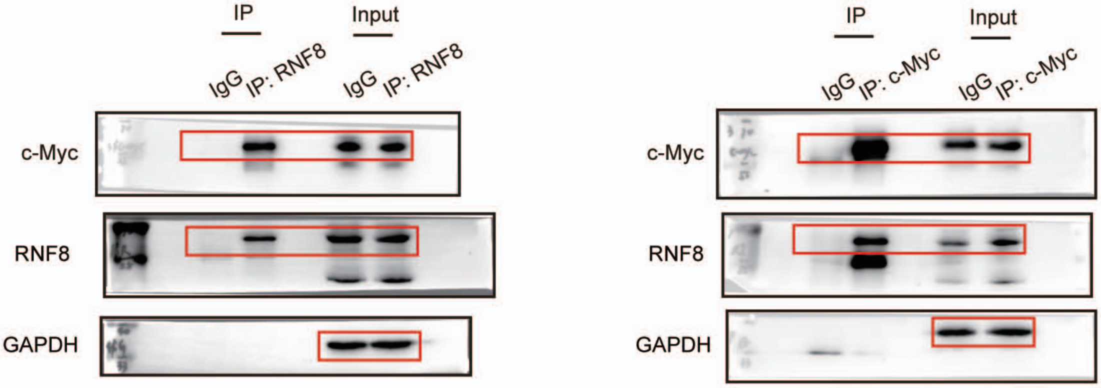


Figure 3C

# Original data file 2

A

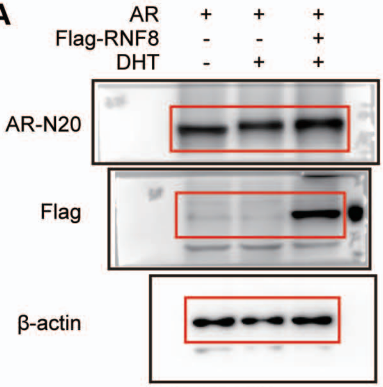


Figure 4A

B

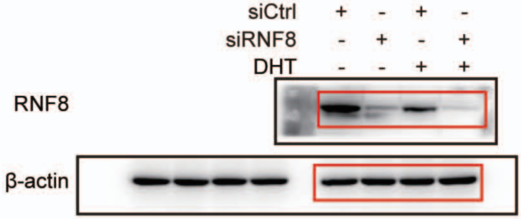


Figure 4B

C

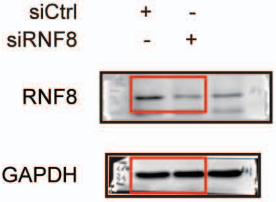


Figure 4C

D

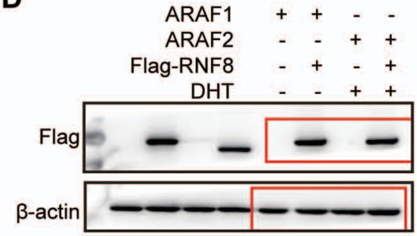


Figure 4D

E

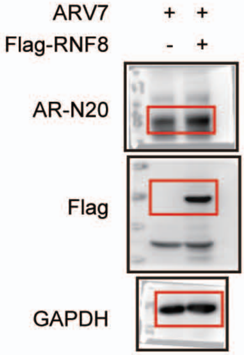


Figure 4E

F

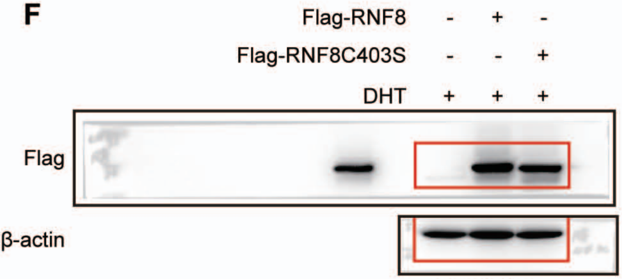


Figure 4F

G

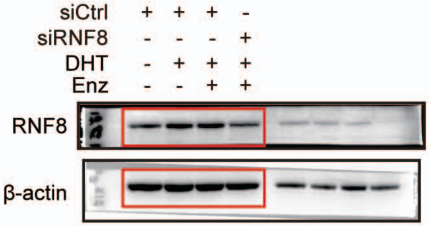


Figure 4G

H

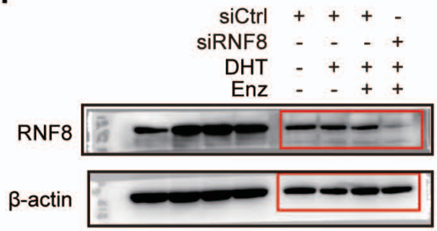


Figure 4H

I

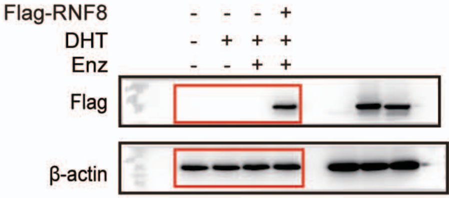


Figure 4I

## Original data file 3

**A**

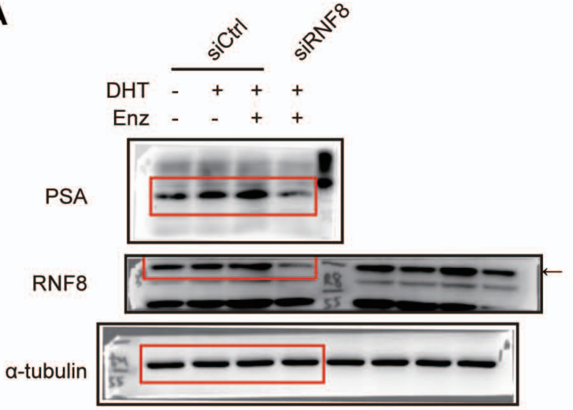


Figure 5F

**B**

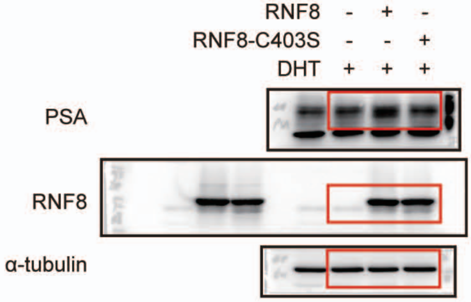


Figure 5H

**C**

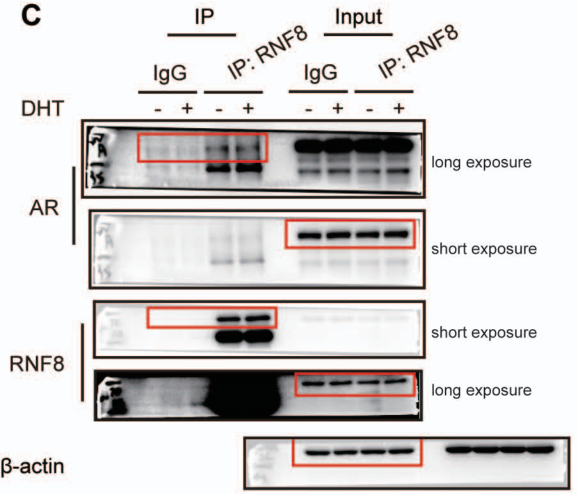


Figure 6A

**D**

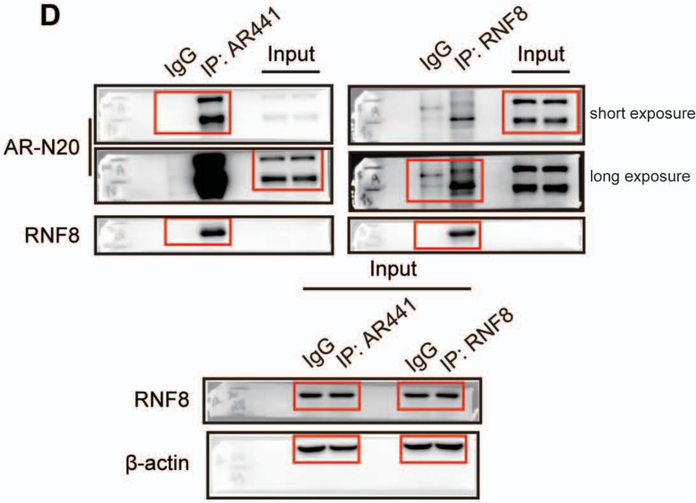


Figure 6B

**E**

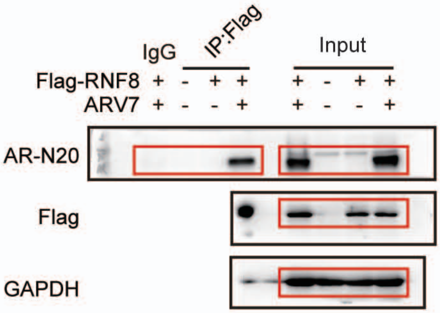


Figure 6D

**F**

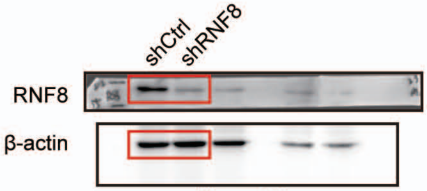


Figure 7E

**G**

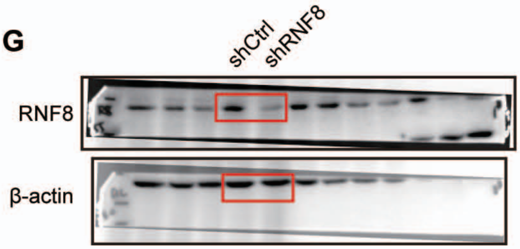


Figure 7M

# Original data file 4

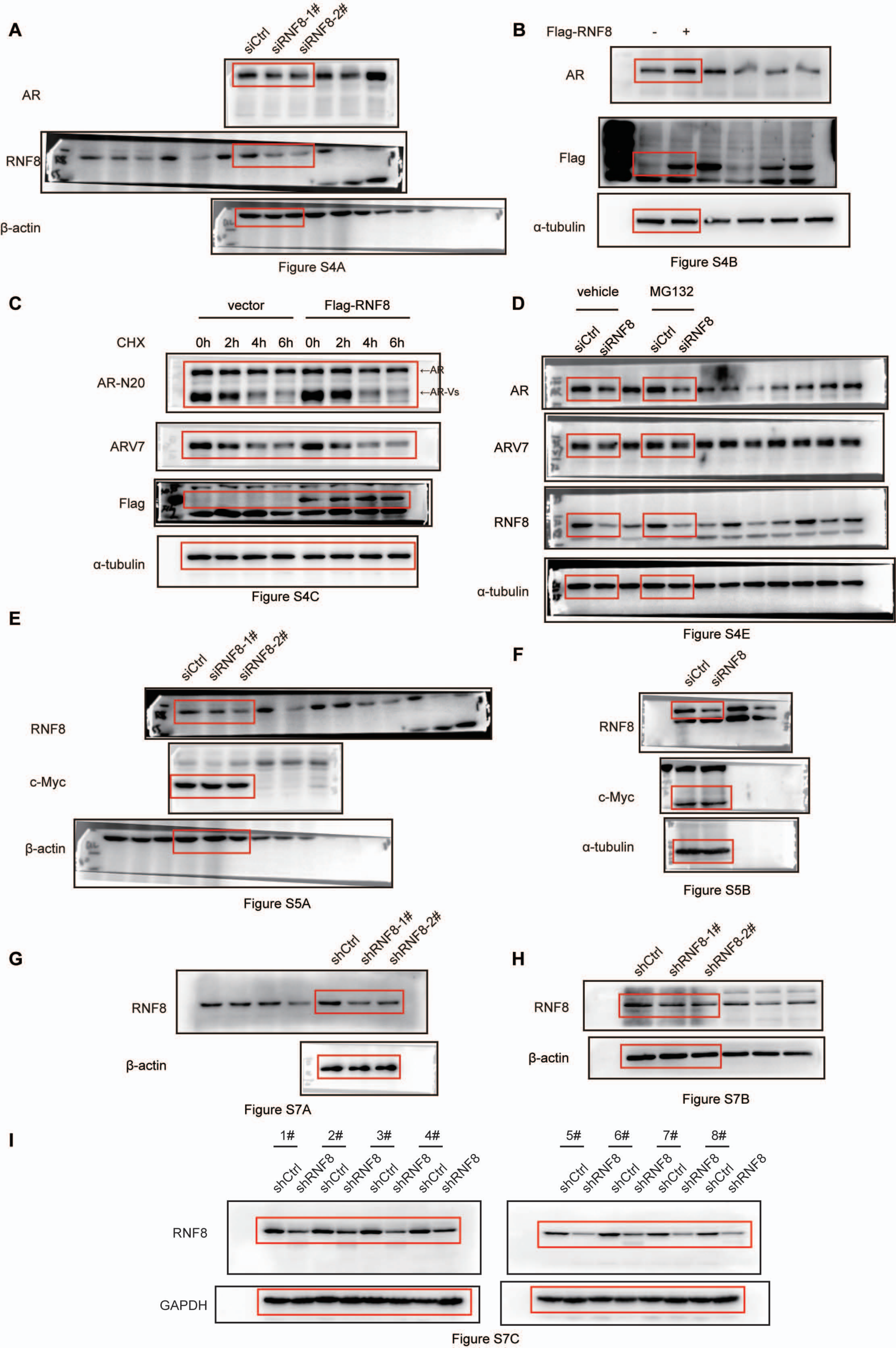

# Original data file 5

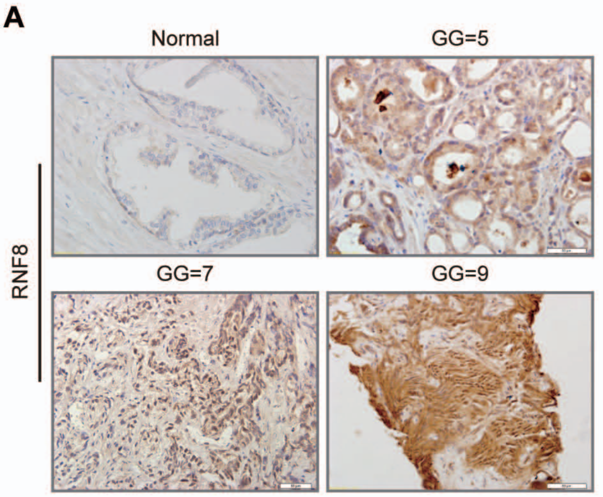


Figure 1C

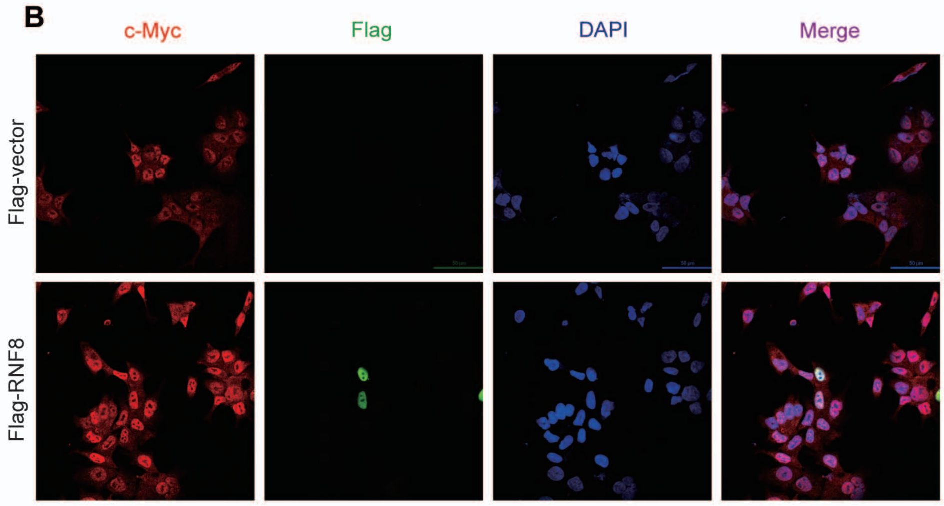


Figure 3A

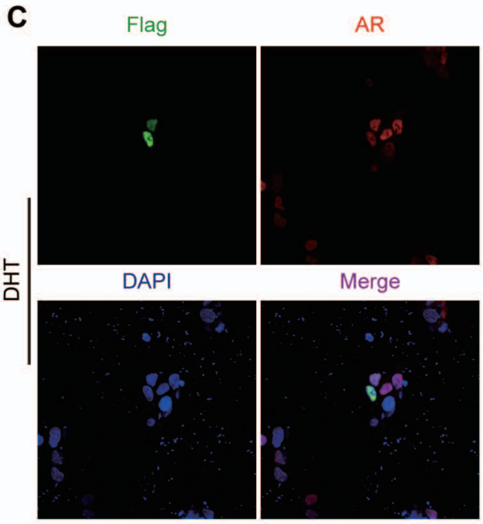


Figure 6B

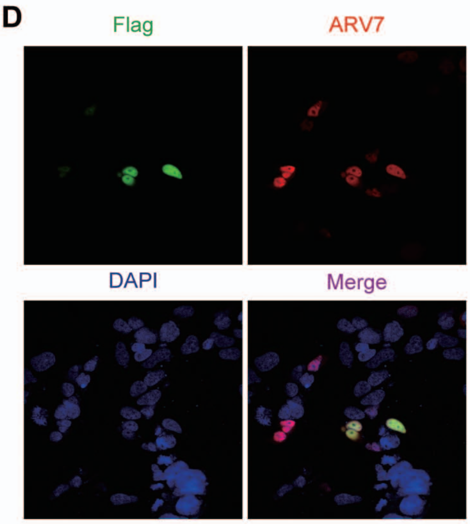


Figure 6E

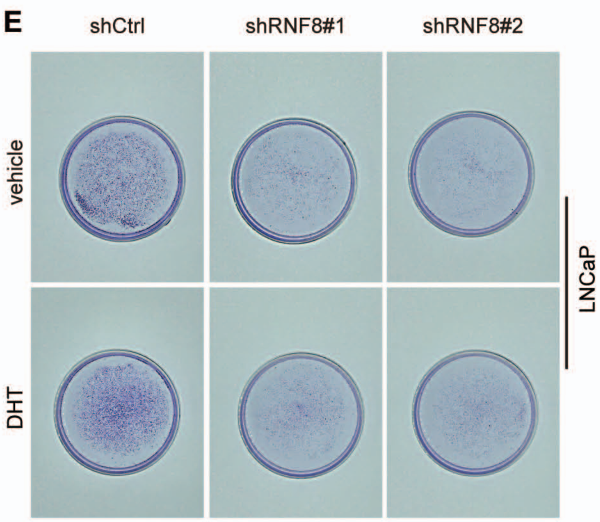


Figure 7A

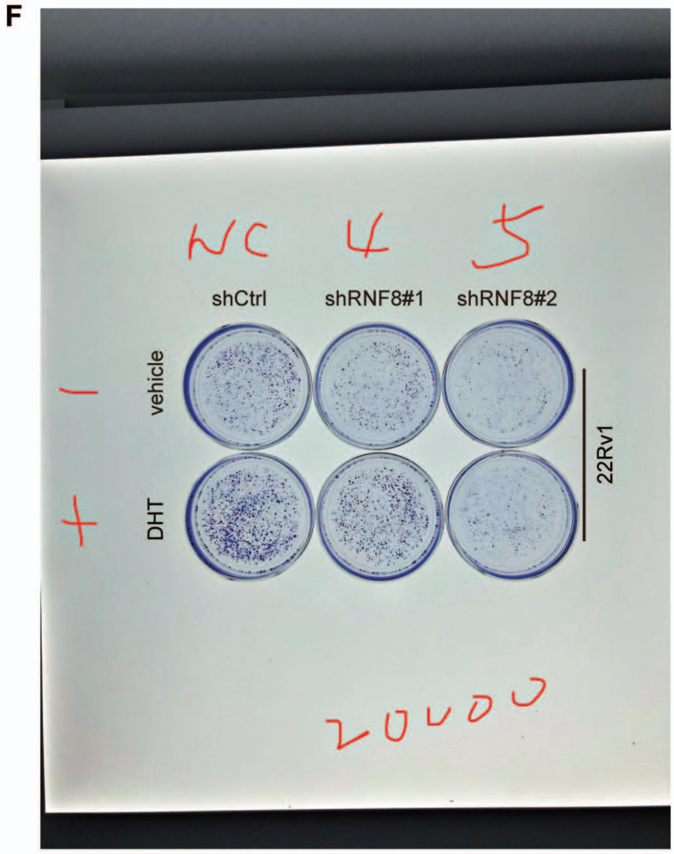


Figure 7B

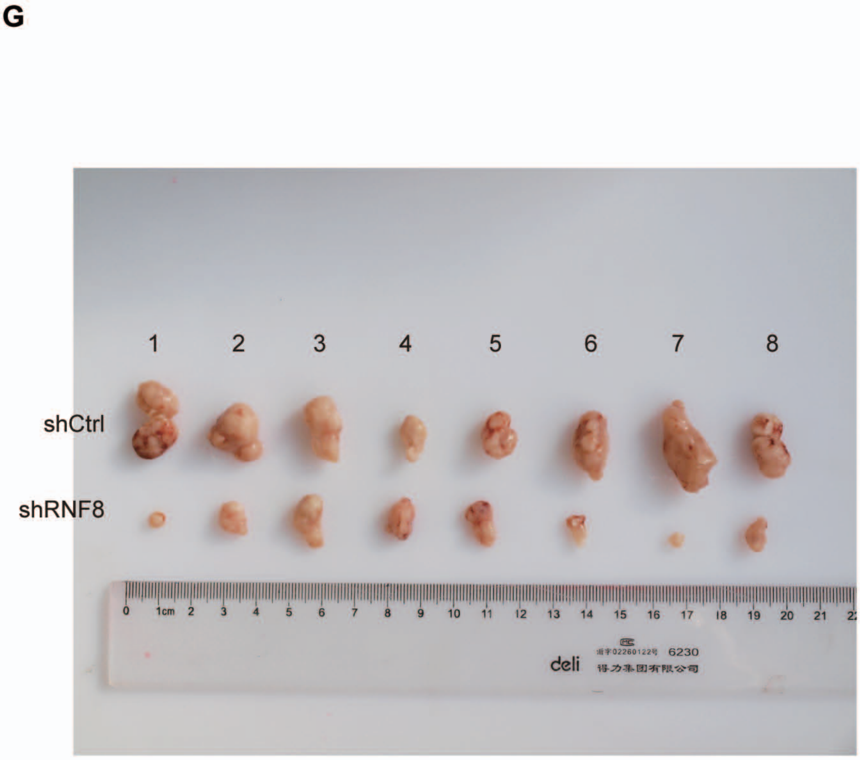


Figure 7F

# Original data file 6

**A**

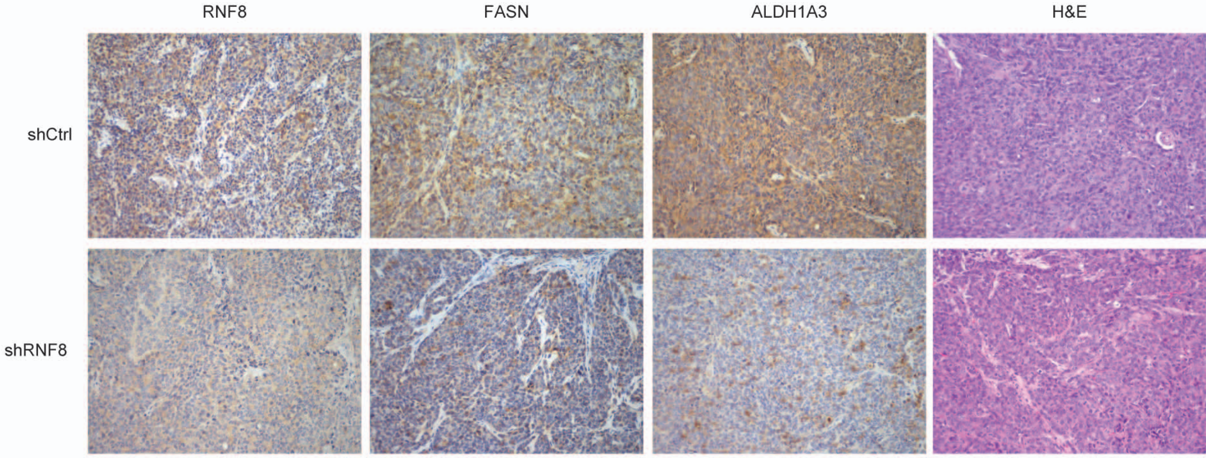


Figure 7I

**B**

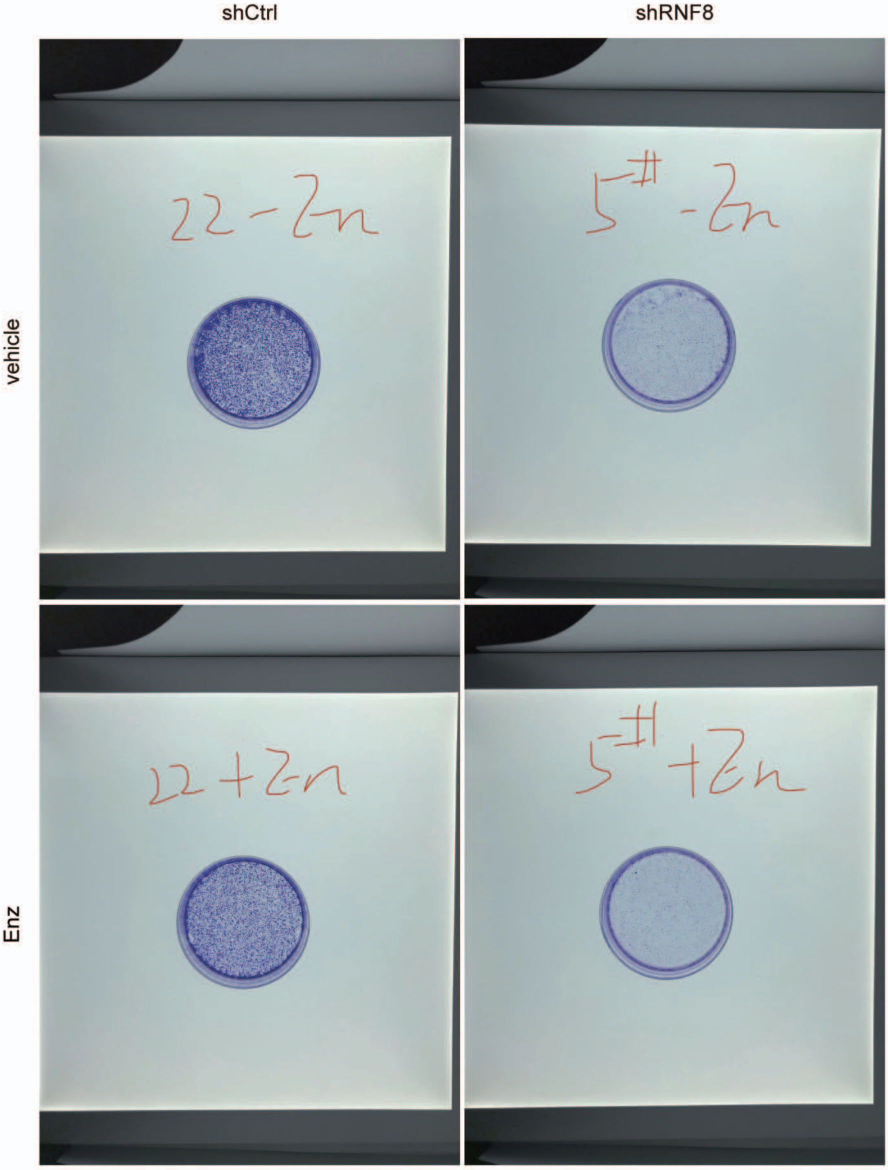


Figure 7K

# Original data file 7

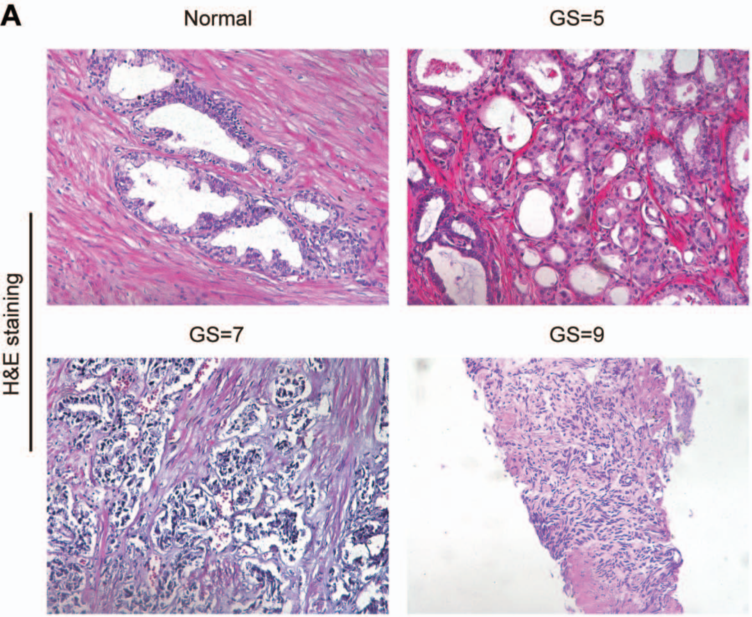


Figure S1A

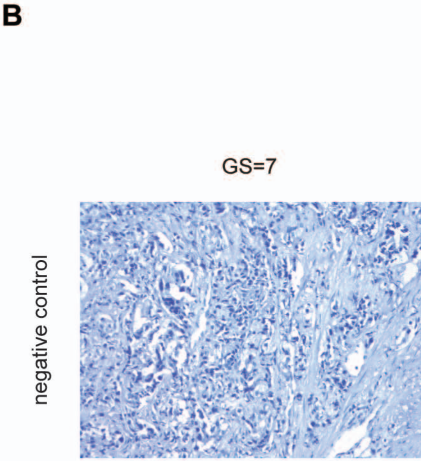


Figure S1B

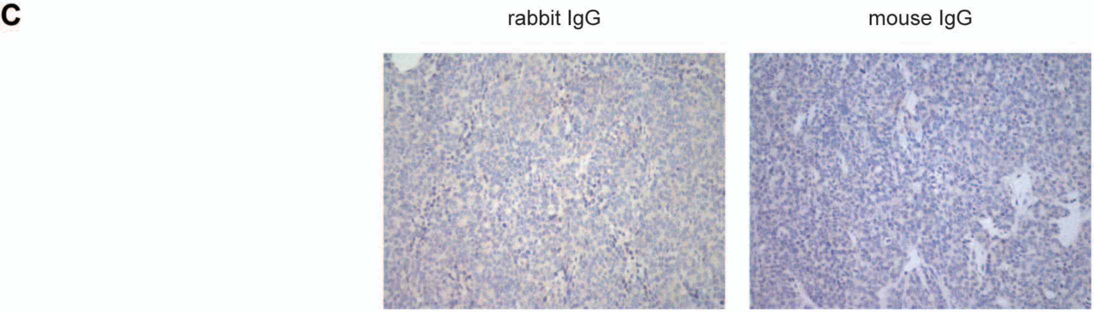


Figure S1C
